# CES1‐Triggered Liver‐Specific Cargo Release of CRISPR/Cas9 Elements by Cationic Triadic Copolymeric Nanoparticles Targeting Gene Editing of PCSK9 for Hyperlipidemia Amelioration

**DOI:** 10.1002/advs.202300502

**Published:** 2023-04-21

**Authors:** Yunfei Zhao, Yun Li, Fan Wang, Xuelan Gan, Tianye Zheng, Mengyue Chen, Li Wei, Jun Chen, Chao Yu

**Affiliations:** ^1^ Chongqing Key Laboratory for Pharmaceutical Metabolism Research, Chongqing Pharmacodynamic Evaluation Engineering Technology Research Center, College of Pharmacy Chongqing Medical University Chongqing 400016 P. R. China; ^2^ Centre for Lipid Research & Key Laboratory of Molecular Biology for Infectious Diseases (Ministry of Education) Institute for Viral Hepatitis Department of Infectious Diseases the Second Affiliated Hospital Chongqing Medical University Chongqing 400016 P. R. China

**Keywords:** CES active destabilized strategy, CRISPR/Cas9, hyperlipidemia amelioration, liver fixed‐point release, mRNA delivery, PCSK9

## Abstract

The broad application of clustered regularly interspaced short palindromic repeat (CRISPR)/Cas9 genome editing tools is hindered by challenges in the efficient delivery of its two components into specific cells and intracytoplasmic release. Herein, a novel copolymer for delivery of Cas9‐mRNA/ single‐guide RNA (Cas9‐mRNA/sgRNA) in vitro and vivo, using carboxylesterase‐responsive cationic triadic copolymeric nanoparticles targeted proprotein convertase subtilisin/kexin type 9 (PCSK9) for hyperlipidemia amelioration is reported. A dimethyl biguanide derivative is designed and synthesized to form cationic block, and copolymerization onto prepolymer with propyl methacrylate, to fabricate a triadic copolymer mPEG*‐b‐*P(Met/n‐PMA). The copolymer can self‐assemble with Cas9‐mRNA/sgRNA, indicating the excellent potential of nanoparticles to form a delivery carrier. This vehicle can efficiently release RNA in response to the hepatocytes carboxylesterase for genome editing. It was demonstrated that the mPEG*‐b‐*P(Met/n‐PMA)/Cas9 mRNA/sgRNA nanoparticles effectively accumulated in hepatocytes, lead to the inhibition of PCSK9, and lowered the levels of Low‐density lipoprotein cholesterol and total cholesterol in mouse serum down 20% of nontreatment. Interestingly, the nanoparticles even enable multiple functions in the regulation of blood glucose and weight. This study establishes a novel method to achieve complex CRISPR components stable loading, safe delivery, and fixed‐point release, which expand the application of CRISPR delivery systems.

## Graphical abstract

This work reports a novel triadic copolymer‐based delivery vehicle to deliver Cas9‐mRNA and sgPCSK9 for gene editing both in vitro and vivo. Carboxylesterase‐responsive motifs are introduced in the vehicle, endowing the delivery vehicle enzymatic responsiveness in hepatocyte and disrupt the target PCSK9 gene and achieve a potent therapeutic efficacy for hyperlipidemia.

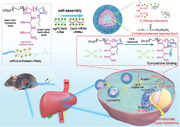

## 1 Introduction

Genome‐editing system is becoming fundamental for biomedical research, gene‐drug development, and even gene therapy.^[^ ^1^ ^]^ Among all the gene editing tools, clustered regularly interspaced short palindromic repeat/Cas9 (CRISPR/Cas9) has shown great advantages in the study of disease treatment strategies owing to its simplicity, flexibility, and promise for a one‐time cure of genetic diseases.^[^ ^2^ ^]^ Several CRISPR/Cas9‐based clinical trials are also underway or about to begin, such as chimeric antigen receptor T‐cell immunotherapy for cancer treatment^[^ ^3^ ^]^ and gene‐editing therapies for targeting the liver for lipid‐lowering therapy.^[^ ^4^ ^]^ However, the safe and effective delivery and release of CRISPR/Cas9 components into target organs, cells, and the nucleus remains a major challenge in genome editing‐mediated disease treatment, which will directly affect the success and therapeutic applications of this system.^[^ ^5^ ^]^

Liposomes, polymers, and inorganic nanoparticles have shown advantages in CRISPR/Cas9 delivery.^[^ ^6^ ^]^ Compared with liposomes and inorganic nanoparticles, polymers show superior modularization and ease of functionalization and can be easily prepared from synthetic monomers or prefabricated polymers, which can precisely control the various properties of nanoparticles during preparation.^[^ ^7^ ^]^ Polymers also have variable drug delivery abilities, allowing polymer nanoparticles to deliver different drugs, including compounds with different molecular weights, such as small molecules, biomacromolecules, and proteins,^[^ ^8^ ^]^ which can meet the demands of different CRISPR/Cas9 codelivery forms (Cas9‐mRNA/sgRNA, plasmid, and Cas9/sgRNA ribonucleoprotein). Thus, they are considered excellent delivery platforms for CRISPR/Cas9. In addition, studies on CRISPR/Cas9 polymers that affect gene delivery and editing processes have also attracted wide interest, including the type of surface charge,^[^ ^9^ ^]^ degree of PEGylation,^[^ ^10^ ^]^ type of CRISPR/Cas9, and location of targeted tissues.^[^ ^11^ ^]^

Cationic copolymers dominate CRISPR/Cas9 delivery because of the high loading potential of CRISPR/Cas9 components and cellular uptake efficiency through electrostatic interactions, with the advent of reversible addition‐fragmentation chain transfer (RAFT) polymerization.^[^ ^12^ ^]^ Several cationic copolymeric carriers have been developed for siRNA delivery.^[^ ^13^ ^]^ However, codelivering larger Cas9‐mRNA molecules or Cas9‐protein with short sgRNAs remains challenging.^[2b]^ More importantly, cationic moieties tend to cause cytotoxicity owing to their interference with cell membrane integrity.^[^ ^14^ ^]^ To date, nearly no cationic polymer nanomaterials have been approved for clinical use by the Food and Drug Administration. Designing and synthesizing innocuous cationic copolymeric delivery systems to resolve these issues is necessary.

Metformin is the first‐line pharmacological treatment for type 2 diabetes, and this cationic drug has shown efficacy and a good safety profile.^[^ ^15^ ^]^ Recent reports have shown that polymers functionalized with guanidinium groups can readily adhere to nucleic acids via Gu^+^/PO_3_^4−^ electrostatic and hydrogen bond interactions.^[^ ^16^ ^]^ Therefore, metformin may be an ideal polymeric monomer for designing safe cationic gene carriers with intrinsic hypoglycemic activity. However, these interactions augment the stability between nucleic acids and guanidinium groups and usually result in poor intracellular release, which consequently leads to inefficient performance.^[^ ^17^ ^]^ Resolving the issue of release while maintaining the stability was also a desirable goal in our study.

Carboxylesterase (CES) is an esterase enzyme family member that hydrolyzes carboxylesterase into its corresponding alcohol and carboxylic acid. Carboxylesterase 1 (CES1) and carboxylesterase 2 (CES2) are two dominant enzymes involved in xenobiotic metabolism. Although CES1 and CES2 share some sequence homology, they exhibit different distributions and substrate specificities.^[^ ^18^ ^]^ For example, CES1 is abundantly expressed in the liver, whereas CES2 is primarily expressed in the small intestine and colon.^[^ ^19^ ^]^ In addition, CES1 preferentially metabolizes ester bonds, which contain a small alcohol group and a large acyl group. In contrast to CES1, CES2 preferentially hydrolyzes esters containing a relatively large alcohol group and a small acyl group.^[^ ^20^ ^]^ Therefore, we hypothesized that incorporating a simple CES1‐active propyl ester monomer in our cationic copolymer would achieve multiple effects. First, we added a hydrophobic force to the cationic copolymer to enable it to effectively encapsulate nucleic acids. Second, reducing some of the positive charges could further reduce the potential cytotoxicity. Third, this structure assists cationic copolymers in selectively releasing RNA from liver cells. Fourth, after activation by CES1, the propyl ester hydrolyzes into the corresponding alcohol and carboxylic acid and emits a negative charge. This negative charge can competitively interfere with the electrostatic and hydrogen bond interactions between biguanides and nucleic acids, promoting further release of nucleic acids.

**Figure 1:**
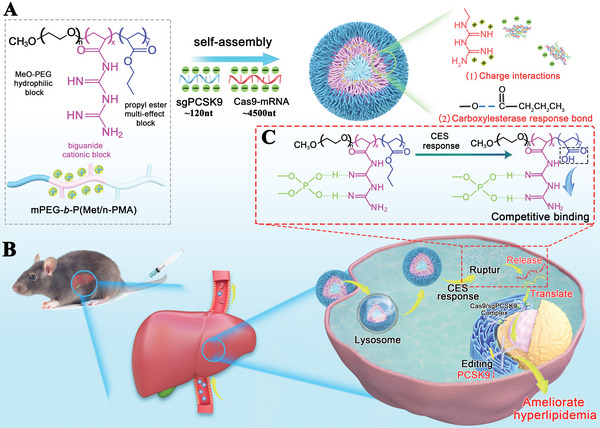
Schematic diagram of using mPEG‐*b*‐P(Met/n‐PMA) for Cas9‐mRNA/sgPCSK9 delivery for hyperlipidemia amelioration. A) The self‐assembly process of mPEG‐*b*‐P(Met/n‐PMA)/Cas9‐mRNA/sgPCSK9. B) Intracellular esterase‐sensitive release hierarchy of mPEG‐*b*‐P(Met/n‐PMA)/Cas9‐mRNA/sgPCSK9 for hyperlipidemia amelioration. C) Mechanisms of esterase‐induced Cas9‐mRNA/sgPCSK9 release. In the presence of hepatocyte esterase, the hydrophobic propyl methacrylate ester is converted into hydrophilic hydroxyl groups. This process first disintegrates mPEG‐*b*‐P(Met/n‐PMA)/Cas9‐mRNA/sgPCSK9 by reducing the hydrophobic stabilization force and subsequently interferes with the electrostatic bond interactions, resulting in effective Cas9‐mRNA/sgPCSK9 release.

Herein, we designed and fabricated a liver CES‐activated dimethyl biguanide triadic copolymer (mPEG*‐b‐*P(Met/n‐PMA), also displayed as PEG‐PMet in the following figures) for the first time. Guanidine and propyl ester monomers were copolymerized onto methoxy PEG‐modified 4‐cyano‐4‐(phenyl thioformyl sulfur) valeric acid (mPEG‐CPADN) using RAFT polymerization. mPEG is another ideal choice for adding hydrophilic force to reduce the toxicity of cationic copolymers because it is commonly used to modify cationic copolymers to reduce protein adsorption and improve nucleic acid transfection efficiency.^[^ ^21^ ^]^ PEG composed of a comb‐like polymer does not exhibit any significant toxic effects.^[^ ^22^ ^]^As shown in **Figure** **1**, we selected the liver proprotein convertase subtilisin/kexin type 9 (PCSK9) as a therapeutic target to demonstrate the application of the copolymer as a nanocarrier for Cas9‐mRNA/sgPCSK9 delivery to reduce plasma low‐density lipoprotein‐ cholesterol (LDL‐C) levels and ameliorate hyperlipidemia. Interestingly, in addition to lipid‐lowering effects, we found a dual effect of this strategy in regulating blood glucose and weight.

## 2 Results and Discussion

### 2.1 Macromolecule Design and Synthesis

**Figure 2:**
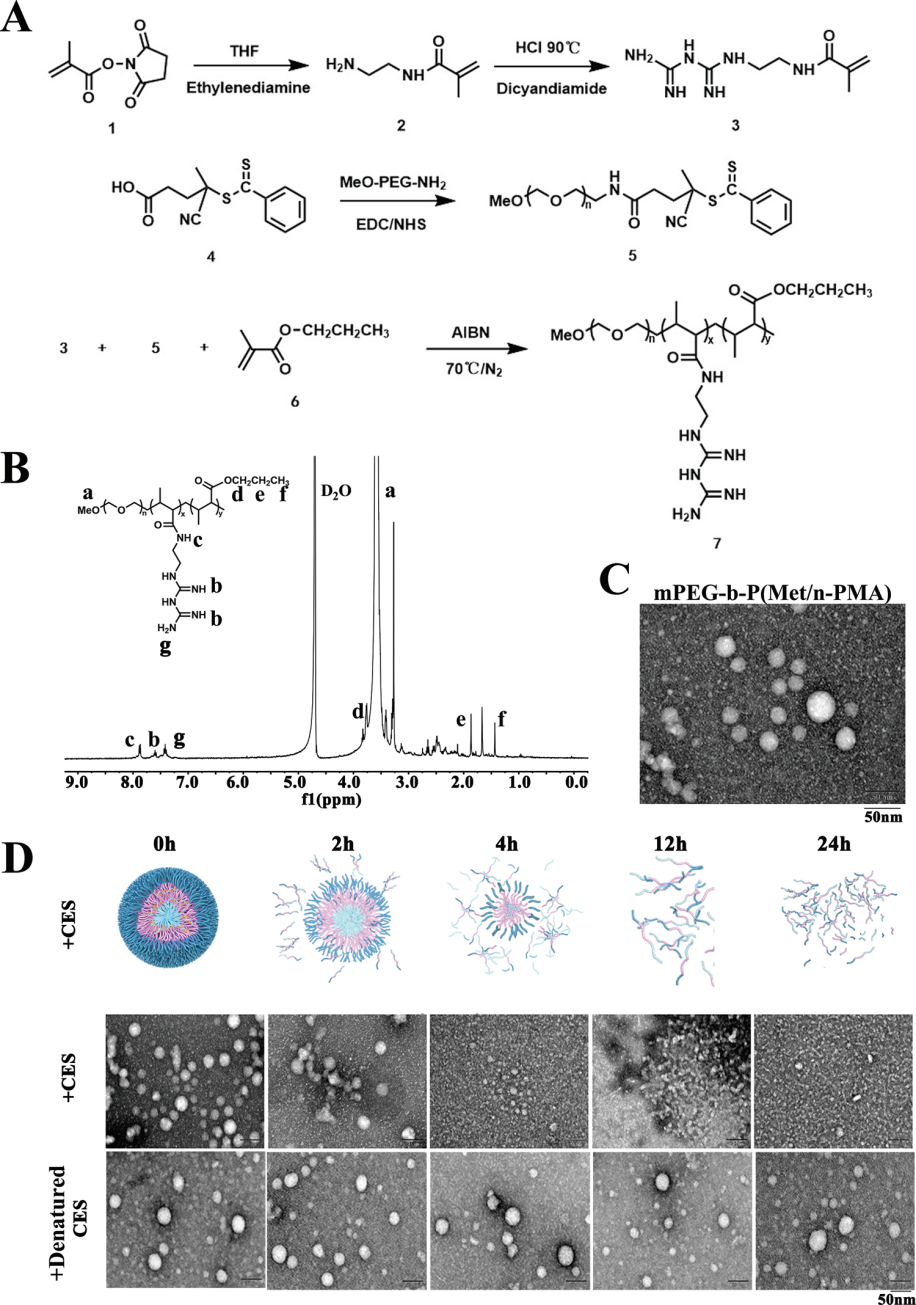
Formation, characterization, and disintegration of mPEG‐*b*‐P(Met/n‐PMA). A) Synthesis of mPEG‐*b*‐P(Met/n‐PMA). B) 1H‐NMR spectrum of mPEG‐*b*‐P(Met/n‐PMA) (D_2_O, 600 MHz). C) TEM image of mPEG‐*b*‐P(Met/n‐PMA). Scale bar: 50 nm. D) Typical TEM images of mPEG‐*b*‐P(Met/n‐PMA) assemblies under 30 U mL^−1^ CES or denatured esterase. Scale bar: 50 nm.

In this study, the mPEG‐CPADN chain‐transfer reagent (Compound 5) was first synthesized (**Figure** **2**A). Subsequent RAFT copolymerization of N‐(2‐(3‐carbamimidoylguanidino)ethyl)methacrylamide (Compound 3) and *n*‐propyl methacrylate (Compound 6) monomers afforded mPEG*‐b‐*P(Met/n‐PMA) using mPEG‐CPADN and azodiisobutyronitrile (AIBN) as macroinitiators. The synthesis processes are described in the Experimental Section and are shown in Figure 2A. The compound structures were confirmed using ^1^H nuclear magnetic resonance (^1^H‐NMR), ^13^C‐NMR, and infrared spectroscopy (Figure 2B and Figures S1–S4, Supporting Information). Further analysis of the ultraviolet (UV) spectrum and gel permeation chromatography showed that the molecular weight of mPEG*‐b‐*P(Met/n‐PMA) was ≈10 000 MW with an UV absorption peak at 305 nm (Figure S5, Supporting Information).

### 2.2 Characterizations and CES Disintegration of mPEG*‐b‐*P(Met/n‐PMA)

Transmission electron microscopy (TEM) showed that the size of mPEG*‐b‐*P(Met/n‐PMA) was ≈30–50 nm (Figure 2C). mPEG*‐b‐*P(Met/n‐PMA) was designed to be esterase‐sensitive. Figure 2D displays the disintegration diagram, and TEM and dynamic light scattering (DLS) (Figure S6, Supporting Information) results showed the in vitro disintegration of mPEG*‐b‐*P(Met/n‐PMA) in the presence of liver CES or denatured liver CES. In addition, mPEG*‐b‐*P(Met/n‐PMA) displayed good stability within 24 h of incubation with mouse serum (Figure S7, Supporting Information).

### 2.3 Molecular Dynamics Simulation of RNA Loading Capacity

**Figure 3:**
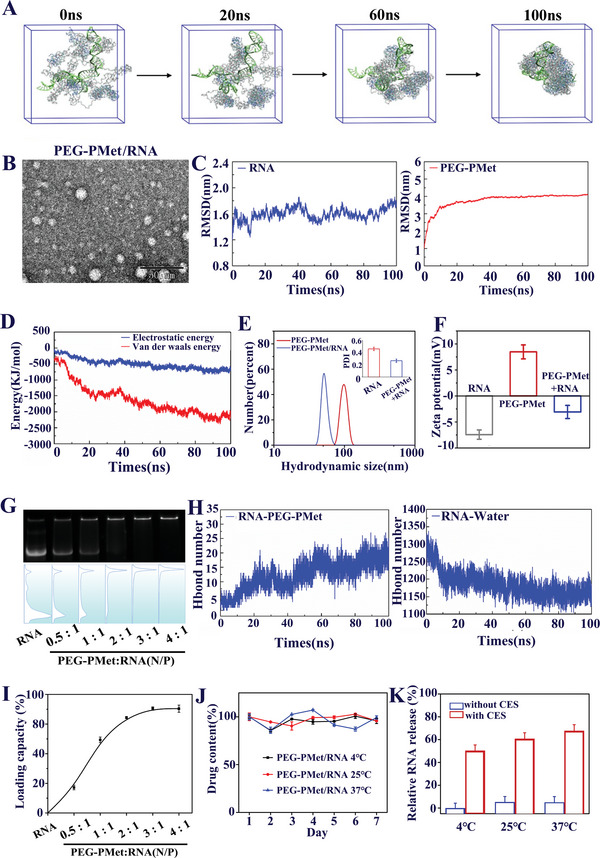
RNA loading and release capacity of mPEG‐*b*‐P(Met/n‐PMA). A) Molecular dynamics simulation of conformation change after mPEG‐*b*‐P(Met/n‐PMA) combines with RNA. B) Root mean square deviation (RMSD) of the mPEG‐*b*‐P(Met/n‐PMA)/RNA complex. (Blue: RNA; Red: mPEG‐*b*‐P(Met/n‐PMA)). C) Energy simulation of the change in the interaction between mPEG‐*b*‐P(Met/n‐PMA) and RNA. D) Change in the H‐bond number between RNA and mPEG‐*b*‐P(Met/n‐PMA) or RNA and water. E) TEM image of mPEG‐*b*‐P(Met/n‐PMA)/RNA. Scale bar: 50 nm. F) DLS size profile and PDI image of mPEG‐*b*‐P(Met/n‐PMA) and mPEG‐*b*‐P(Met/n‐PMA)/RNA. Bars represent the mean ± SD (*n* = 3). G) Zeta potential of RNA, mPEG‐*b*‐P(Met/n‐PMA), and mPEG‐*b*‐P(Met/n‐PMA)/RNA in water. Bars represent the mean ± SD (*n* = 3). H) Detection of RNA binding ability of different mole ratios of mPEG‐*b*‐P(Met/n‐PMA) by gel electrophoresis. I) Loading capacity of mPEG‐*b*‐P(Met/n‐PMA) for RNA in mass. Bars represent the mean ± SD (*n* = 3). J) Drug content of RNA encapsulated in mPEG‐*b*‐P(Met/n‐PMA) at 4, 25, and 37 °C for one week. Bars represent the mean ± SD (*n* = 3). K) RNA release from mPEG‐*b*‐P(Met/n‐PMA) following esterase treatment as determined via UV spectroscopy. Bars represent the mean ± SD (*n* = 3).

To gain insights into the mechanism and degree of self‐assembly between mPEG*‐b‐*P(Met/n‐PMA) and RNA (mPEG*‐b‐*P(Met/n‐PMA)/RNA), the molecular dynamics (MD) simulation method was used to simulate the formation processes of mPEG*‐b‐*P(Met/n‐PMA)/RNA. In a typical simulation process, energy‐optimized RNA and mPEG*‐b‐*P(Met/n‐PMA) 3D models were constructed (Figure S8, Supporting Information). Then, eight mPEG*‐b‐*P(Met/n‐PMA) molecules and one RNA molecule were solvated into a 15 cm × 15 cm × 15 cm cubic water box, which showed that all molecules self‐assembled into spherical particles after 100 ns (**Figure** **3**A). The results indicated that oxygen atoms and nitrogen atoms in mPEG*‐b‐*P(Met/n‐PMA) form hydrogen bonds with RNA, while methylene constitutes *σ*–*π* stacking with RNA (Figure S9, Supporting Information). Figure 3B shows the changes in the root mean square deviation (RMSD) of RNA and mPEG*‐b‐*P(Met/n‐PMA). The RMSD of RNA fluctuates slightly in the entire process of simulation, indicates that the RNA molecule is relatively stable in the entire process of simulation. The RMSD of mPEG*‐b‐*P(Met/n‐PMA) changed significantly at the beginning of the simulation and tended to stabilize, indicating that mPEG*‐b‐*P(Met/n‐PMA) and RNA could assemble to form stable nanoparticles. Next, the energy magnitude between mPEG*‐b‐*P(Met/n‐PMA) and RNA was explored using the GROMACS energy module (Figure 3C). This result suggests that with the continuous aggregation of the complex, the electrostatic (originating from hydrogen bond interactions) and van der Waals energies were enhanced. As shown in Figure 3D, the number of hydrogen bonds increased between mPEG*‐b‐*P(Met/n‐PMA) and RNA, while that between RNA and water decreased, indicating that hydrogen bonding is the major driving force for the aggregation between mPEG*‐b‐*P(Met/n‐PMA) and RNA. In the self‐assembly of PEG‐PMet and RNA, the area exposed to the solvent gradually decreases; therefore, the solvent‐accessible surface area (SASA) can be used to evaluate the self‐assembly compactness of the spherical particles. Figure S10 (Supporting Information) shows that the SASA value of the PEG‐PMet/RNA complex decreased and tended to stabilize, indicating that PEG‐PMet and RNA aggregated and self‐assembled into stable micelles. In addition, the SASA remained stable in the later stage of the simulation, indicating that the cluster structure did not dissociate owing to the influence of the solvent.

### 2.4 RNA Loading Capacity and Release Profile

TEM demonstrated that the conventional single hydrogen bonding‐stabilized mPEG*‐b‐*P(Met/n‐PMA)/RNA complex was 20–30 nm in size (Figure 3E), which was smaller than that of single mPEG*‐b‐*P(Met/n‐PMA) (Figure 3F), because the increasing number of stabilizing interaction forces endowed high RNA condensation ability. Furthermore, the average zeta potential of mPEG*‐b‐*P(Met/n‐PMA)/RNA was increased to −3 mV compared with that of single mPEG*‐b‐*P(Met/n‐PMA) and single RNA (Figure 3G). Then, we further discussed the diameters and zeta potentials of mPEG*‐b‐*P(Met/n‐PMA)/RNA nanoparticles, which were prepared at different N/P ratios from 0.5 to 4 (Figure S11A, Supporting Information). With an increase in the N/P ratio, the size of mPEG*‐b‐*P(Met/n‐PMA)/RNA decreased gradually, possibly due to the enhanced cooperative interactions between the cationic mPEG*‐b‐*P(Met/n‐PMA) polymer and anionic RNA. Figure S11B (Supporting Information) shows the zeta potentials of mPEG*‐b‐*P(Met/n‐PMA)/RNA at different N/P ratios. At an N/P ratio of 0.5:1, the mPEG*‐b‐*P(Met/n‐PMA)/RNA exhibited the highest negative zeta potential. Upon the addition of increasing amounts of the cationic polymer, the zeta potential values of the formed nanoparticles increased proportionally. At an N/P ratio of 4:1, a positive zeta potential value was obtained. Gel electrophoresis and UV spectroscopy demonstrated that the polymer effectively loaded RNA at an N/P ratio of 3:1 (Figure 3H and Figure S12, Supporting Information), and the entrapment rate reached 70% (Figure 3I). The release profile of mPEG*‐b‐*P(Met/n‐PMA)/RNA was investigated by monitoring the increase in the number of active complexes in the supernatant. To exclude the possibility of the self‐release of mPEG*‐b‐*P(Met/n‐PMA)/RNA, we monitored the stability of mPEG*‐b‐*P(Met/n‐PMA)/RNA at different temperatures. As shown in Figure 3J, the RNA encapsulated into mPEG*‐b‐*P(Met/n‐PMA) did not degrade within a week. Under these conditions, esterase was added to mPEG*‐b‐*P(Met/n‐PMA)/RNA, and the UV spectrum could detect the release of RNA (Figure 3K), suggesting that the release of RNA was triggered by esterase.

### 2.5 Intracellular Delivery

**Figure 4:**
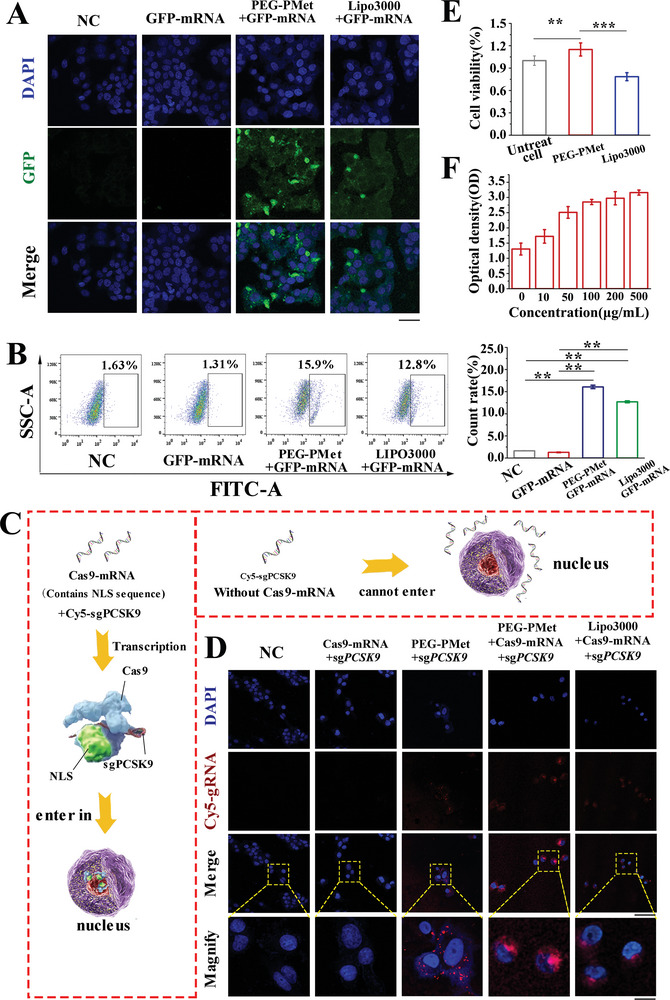
Intracellular delivery and degradation of mPEG*‐b‐*P(Met/n‐PMA) loaded with mRNA and gRNA. A) CLSM analysis of GFP‐mRNA cellular uptake and transcript accumulation in HepG2 cells, including negative control (NC), GFP‐mRNA, mPEG*‐b‐*P(Met/n‐PMA)/GFP‐mRNA, and Lipo3000/GFP‐mRNA (Lipofectamine 3000/GFP‐mRNA). Green: GFP, Blue: Nucleus. Scale bar: 50 µm. B) FCM analysis of GFP‐mRNA cellular uptake and transcript accumulation in HepG2 cells. (**P < 0.01, Statistical analysis was determined using one‐way ANOVA). Bars represent the mean ± SD (*n* = 3). C) In vitro cell viability of HepG2 cells treated with Lipo3000, mPEG*‐b‐*P(Met/n‐PMA), and D) different concentrations of mPEG*‐b‐*P(Met/n‐PMA) determined by CCK8. (***P < 0.001, **P < 0.01, determined using one‐way ANOVA), Bars represent mean ± SD (*n* = 3). E) Schematic diagram of intracellular transmission of Cas9‐mRNA/sgPCSK9. After Cas9‐mRNA‐containing nuclear localization signal (NLS) sequences were transcribed in the cytoplasm, Cas9/sgPCSK9 ribonucleoprotein, mediated by the NLS, was further imported into nuclei. F) CLSM analysis of Cas9‐mRNA/sgPCSK9 cellular uptake, transcription, and accumulation in the nucleus, including NC, Cas9‐mRNA/sgPCSK9, mPEG*‐b‐*P(Met/n‐PMA)/sgPCSK9, mPEG*‐b‐*P(Met/n‐PMA)/Cas9‐mRNA/sgPCSK9, and Lipo3000/Cas9‐mRNA/sgPCSK9. Red: Cy5‐sgPCSK9, Blue: Nucleus. Scale bars: 50 and 20 µm magnification.

We evaluated the intracellular delivery of green fluorescent protein (GFP) and its mRNA (GFP‐mRNA), which were encapsulated into mPEG*‐b‐*P(Met/n‐PMA), using HepG2, HeLa, HEK293, and A549 cells (Figure S13, Supporting Information). Confocal laser scanning microscopy (CLSM) analysis indicated that HepG2 cells showed much higher internalization efficiency than other cells because the esterase content was different in different cells. (Esterase in HepG2 cells is more highly activated than that in HeLa cells, while HEK293 and A549 cells are almost non‐expressed^[^ ^23^ ^]^). mPEG*‐b‐*P(Met/n‐PMA)‐delivered GFP mRNA showed higher fluorescence than GFP, GFP‐mRNA, and mPEG*‐b‐*P(Met/n‐PMA)/GFP. In addition, mPEG*‐b‐*P(Met/n‐PMA)/GFP‐mRNA showed a much higher internalization efficiency than the other controls (negative control, GFP‐mRNA, and Lipo3000/GFP‐mRNA) (**Figure** **4**A). FACS revealed FITC positivity, which was ≈15‐fold higher than that in the negative control and GFP‐mRNA groups (Figure 4B). Furthermore, sgPCSK9 was labeled with Cy5 (Cy5‐sgPCSK9), and Cas9‐mRNA containing the nuclear localization signal (NLS) domain was encapsulated into mPEG*‐b‐*P(Met/n‐PMA) to evaluate the nuclear import of mPEG*‐b‐*P(Met/n‐PMA)/Cas9‐mRNA/sgPCSK9 (Figure 4C). sgPCSK9 transcription was successfully synthesized and verified by sequence analysis (Figure S14, Supporting Information). According to CLSM analysis (Figure 4D and Figure S15, Supporting Information), efficient internalization of mPEG*‐b‐*P(Met/n‐PMA)/Cas9‐mRNA/Cy5‐sgPCSK9 and Lipo3000/Cas9‐mRNA/Cy5‐sgPCSK9 was observed, which escaped from lysosomes, and were distributed in and around the nuclei. The mechanism of mPEG‐b‐P(Met/n‐PMA)‐mediated lysosomal escape may cause by the ATP‐depleting dependent way of polymetformin‐based polycationic micelles.^[^ ^24^ ^]^ As a control, mPEG*‐b‐*P(Met/n‐PMA), containing only Cy5‐sgPCSK9, exhibited negligible cytosolic delivery and nuclear import. In a cytotoxicity test using the CCK8 method, mPEG*‐b‐*P(Met/n‐PMA) was also shown to facilitate cell viability, while Lipo3000 showed cytotoxicity (Figure 4E). The promotion of cell viability by mPEG*‐b‐*P(Met/n‐PMA) increased with increasing dose, and even the dose of 500 µg mL^−1^ showed no cytotoxicity (Figure 4F). As a metformin derivative, we speculated that the cell proliferation effect of mPEG*‐b‐*P(Met/n‐PMA) might result from its biguanide structure. Metformin can facilitate the proliferation, migration, and differentiation of cells in vitro to a certain degree by pathways, such as ERK/AMPK.^[8a,25]^

### 2.6 Evaluation of PCSK9 Gene Editing In Vitro

**Figure 5:**
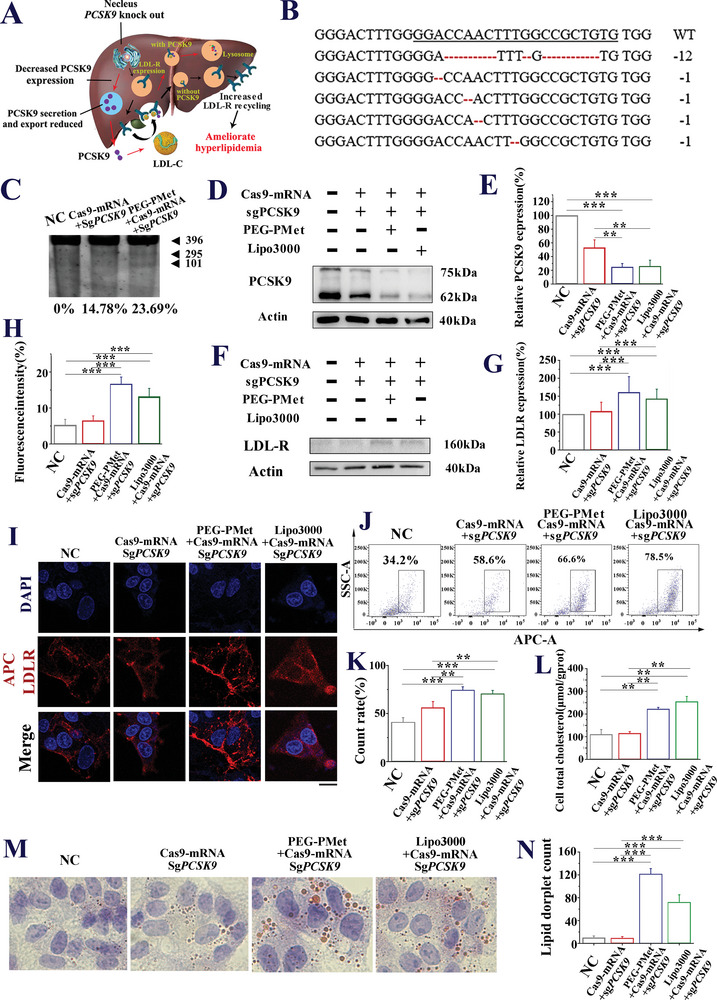
Effect of mPEG‐b‐P(Met/n‐PMA)/Cas9‐mRNA/sgPCSK9‐mediated genome editing in vitro. A) Molecular mechanism by which PCSK9 knockdown ameliorates hyperlipidemia. B) High‐throughput sequencing of the PCSK9 genomic locus targeted by mPEG‐*b*‐P(Met/n‐PMA)/Cas9‐mRNA/sgPCSK9 in HepG2 cells. Five out of 30 sequencing results showed indels around the sgRNA targeting domain. The underline represents the nucleotide sequence of sgRNA targeting PCSK9. The number of deletions compared to the wild type is shown on the right. C) T7E1 assay of HepG2 cells treated with Cas9‐mRNA/sgPCSK9 and mPEG‐*b*‐P(Met/n‐PMA)/Cas9‐mRNA/sgPCSK9. D) One out of the E) three representative experiments of the PCSK9 protein levels and quantitative data in HepG2 cells were examined by western blotting after treatment with Cas9‐mRNA/sgPCSK9, mPEG‐*b*‐P(Met/n‐PMA)/Cas9‐mRNA/sgPCSK9, and Lipo3000/Cas9‐mRNA/sgPCSK9. F,G) LDLR level on the HepG2 cell membrane according to western blotting analysis, H,I) immunofluorescence image (Red: APC‐LDLR, Blue: Nucleus, Scale bar: 20 µm). J,K) Flow cytometry. L) Total cholesterol levels in HepG2 cells treated under different conditions, including NC, Cas9‐mRNA/sgPCSK9, PEG‐PMet/Cas9‐mRNA/sgPCSK9, and Lipo3000/Cas9‐mRNA/sgPCSK9. M) Morphological examination and N) lipid content of HepG2 cells after transfection with Cas9‐mRNA/sgPCSK9, mPEG‐*b*‐P(Met/n‐PMA)/Cas9‐mRNA/sgPCSK9, and Lipo3000/Cas9‐mRNA/sgPCSK9 (Oil Red O staining, original magnification 400×, analyzed by Image‐Pro Plus). ***P < 0.001, **P < 0.01, Bars represent the mean ± SD (*n* = 3), *P*‐values are calculated using one‐way ANOVA with Bonferroni correction.

Next, we examined the gene editing efficacy of Cas9‐mRNA/sgPCSK9 delivered by different carriers in HepG2 cells. The mechanism by which PCSK9 knockdown ameliorated hyperlipidemia is shown in **Figure** **5**A. High‐throughput sequencing (Figure 5B) and T7E1 assays (Figure 5C) indicated that mPEG*‐b‐*P(Met/n‐PMA)/Cas9‐mRNA/sgPCSK9 induced effective editing of the PCSK9 gene, whereas the control groups induced insignificant editing. Western blotting indicated that mPEG*‐b‐*P(Met/n‐PMA)/Cas9‐mRNA/sgPCSK9 significantly downregulated the expression of PCSK9 (Figure 5D,E) and upregulated that of low‐density lipoprotein receptor (LDL‐R) (Figure 5F,G). Immunofluorescence (Figure 5H,I) and FACS (Figure 5J,K) showed that mPEG*‐b‐*P(Met/n‐PMA)/Cas9‐mRNA/sgPCSK9 improved the brightness of APC, suggesting that the LDL‐R content on the cell membrane surface increased significantly. Two other experimental groups detected an exogenous LDL‐C uptake effect of mPEG*‐b‐*P(Met/n‐PMA)/Cas9‐mRNA/sgPCSK9 in HepG2 cells. The detection of total cholesterol levels (Figure 5L) and Oil Red O staining (Figure 5M,N) revealed that mPEG*‐b‐*P(Met/n‐PMA)/Cas9‐mRNA/sgPCSK9 promoted the uptake of exogenous cholesterol by HepG2 cells. In addition, we used the CRISPR Design server to identify eight sgPCSK9 off‐target sites. Then, we amplified these eight potential off‐target sites from mPEG*‐b‐*P(Met/n‐PMA)/Cas9‐mRNA/sgPCSK9, treated them with HepG2, and evaluated CRISPR/Cas9 editing using the T7 endonuclease I (T7EI) assay. No off‐target cutting was observed at any off‐target site (Figures S16 and S23, Supporting Information).

### 2.7 CES1‐Responsive Effect In Vitro

As shown in Figure S17 (Supporting Information), mPEG*‐b‐*P(Met) (also referred to as PPM in the following figure) was fabricated using RAFT polymerization, in which only the guanidine monomer was polymerized onto mPEG‐CPADN (Figure S17A, Supporting Information). The structure of mPEG*‐b‐*P(Met) was confirmed using ^1^H‐NMR (Figure S17B, Supporting Information). The TEM images (Figure S17C,D, Supporting Information) showed that mPEG*‐b‐*P(Met) did not form nanoparticles, whereas self‐assembly occurred after RNA was added (mPEG*‐b‐*P(Met)/RNA). This might be attributed to the weakening of hydrogen bonds between RNA and water on the formation of new hydrogen bonds between RNA and mPEG*‐b‐*P(Met), which cause the properties of RNA to change from hydrophilic to hydrophobic and introduce hydrophobic interactions.^[^ ^26^ ^]^ UV spectra revealed the RNA loading and release abilities of mPEG*‐b‐*P(Met) (Figure S17E,F, Supporting Information), suggesting that the RNA bound to mPEG*‐b‐*P(Met) could not be triggered by esterase. Western blot assays showed that mPEG*‐b‐*P(Met/n‐PMA)/Cas9‐mRNA/sgPCSK9 showed better effects on PCSK9 and LDL‐R than the nanoparticles that were non‐CES1‐responsive (Figure S17G, Supporting Information). Oil Red O staining further revealed that mPEG*‐b‐*P(Met/n‐PMA)/Cas9‐mRNA/sgPCSK9 showed higher uptake ability for exogenous cholesterol than mPEG‐*b*‐P(Met)/Cas9‐mRNA/sgPCSK9 (Figure S17H,I, Supporting Information).

### 2.8 Biodistribution In Vivo

**Figure 6:**
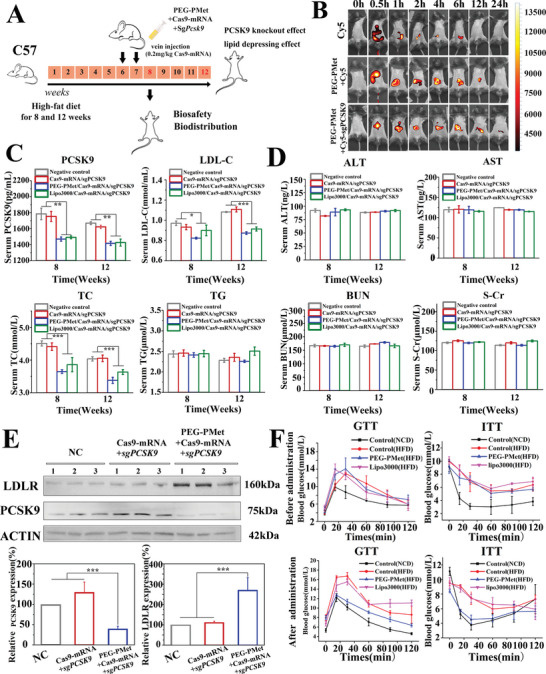
Hyperlipidemia amelioration mediated by mPEG‐*b*‐P(Met/n‐PMA)/Cas9‐mRNA/sgPCSK9 in mice. A) Schematic illustration of hyperlipidemia amelioration in C57BL/6J mice and the treatment schedule for treatment with mPEG‐*b*‐P(Met/n‐PMA)/Cas9‐mRNA/sgPCSK9. B) Luminescence images of C57BL/6J mice following treatment with Cy5, mPEG‐*b*‐P(Met/n‐PMA)/Cy5, and mPEG‐*b*‐P(Met/n‐PMA)/Cy5‐sgPCSK9. C) Serum levels of PCSK9 protein, LDL‐C, TC, and TG in mice 14 or 42 d after administration of mPEG‐*b*‐P(Met/n‐PMA)/Cas9‐mRNA/sgPCSK9 and those in the control groups (*n* = 6 mice for each group). D) Levels of typical hematological parameters of mice, including ALT, AST, BUN, and S‐Cr, 14 or 42 d after administration of mPEG‐*b*‐P(Met/n‐PMA)/Cas9‐mRNA/sgPCSK9 and those in the control groups (*n* = 6 mice for each group). E) Western blot analysis of liver PCSK9 and LDLR levels from mice 42 d after the administration of different formulations, including negative control, Cas9‐mRNA/sgPCSK9, mPEG‐*b*‐P(Met/n‐PMA)/Cas9‐mRNA/sgPCSK9, and Lipo3000/Cas9‐mRNA/sgPCSK9. F) Glucose tolerance tests (GTTs, left) and insulin tolerance tests (ITTs, right) in HFD‐fed mice before and after the administration of mPEG‐*b*‐P(Met/n‐PMA) and Lipo3000 (*n* = 3). ***P < 0.001, **P < 0.01, *P*‐values are calculated using one‐way ANOVA. Bars represent the mean ± SD (*n* = 3).

The three groups of animals received a single dose of Cy5, mPEG*‐b‐*P(Met/n‐PMA)/Cy5, or mPEG*‐b‐*P(Met/n‐PMA)/Cas9‐mRNA/Cy5‐sgPCSK9. As shown in **Figure** **6**B, mPEG*‐b‐*P(Met/n‐PMA)/Cy5 and mPEG*‐b‐*P(Met/n‐PMA)/Cas9‐mRNA/Cy5‐sgPCSK9 showed significant liver accumulation at 0.5 h postinjection, as strong fluorescence was evident in the liver, and a strong fluorescence signal was observed in the liver. 12 h after injection, the fluorescence in the liver showed a gradually decreasing trend. In contrast, the Cy5 group exhibited rapid and complete metabolism as fluorescence was observed in the intestine at 0.5 h and in the kidneys at 2 h. Biodistribution analysis confirmed that mPEG*‐b‐*P(Met/n‐PMA)/Cy5 and mPEG*‐b‐*P(Met/n‐PMA)/Cas9‐mRNA/Cy5‐sgPCSK9 had better accumulation and retention properties than free Cy5. In addition, the ex vivo fluorescence image of the excised organs further confirmed that mPEG*‐b‐*P(Met/n‐PMA)/Cas9‐mRNA/Cy5‐sgPCSK9 showed a higher fluorescence signal in the liver than free fluorescent molecules (Figure S18, Supporting Information).

### 2.9 Lipid‐Lowering Effects of mPEG*‐b‐*P(Met/n‐PMA)/Cas9‐mRNA/sgPCSK9 In Vivo

We evaluated the effects of PCSK9 editing and tested the serum biochemical parameters after 14 and 42 d of mPEG*‐b‐*P(Met/n‐PMA)/Cas9‐mRNA/sgPCSK9 administration in mice (Figure 6A). The mPEG*‐b‐*P(Met/n‐PMA)/Cas9‐mRNA/sgPCSK9‐administered mice showed a PCSK9 level of 1470 pg mL^−1^, which was substantially lower than that in the other groups (Figure 6C). Furthermore, mPEG*‐b‐*P(Met/n‐PMA)/Cas9‐mRNA/sgPCSK9‐administered mice had significantly lower levels of LDL‐C and total cholesterol (TC), with ≈20% reduction, compared to the control group (Figure 6C). There were no significant differences in the high‐density lipoprotein cholesterol (HDL‐C) (Figure S19, Supporting Information), triglyceride (TG) (Figure 6C), alanine aminotransferase (ALT) (Figure 6D), and aspartate aminotransferase (AST) (Figure 6D), urea nitrogen (BUN) (Figure 6D), and creatinine (S‐Cr) levels (Figure 6D) among the four groups, indicating that none of these formulations had any adverse effects on the mice. We assessed the PCSK9 and LDL‐R levels in the liver. As expected, mPEG*‐b‐*P(Met/n‐PMA)/Cas9‐mRNA/sgPCSK9‐administered mice had lower levels of PCSK9 and higher levels of LDLR protein than control mice (Figure 6E). Hematoxylin and eosin (H&E) staining (Figure S20, Supporting Information) of the major organ sections also indicated that there was no observable effect on the liver or other organs.

### 2.10 mPEG*‐b‐*P(Met/n‐PMA) Regulated Weight Gain and Relieves Insulin Resistance

While evaluating the efficacy and safety of mPEG*‐b‐*P(Met/n‐PMA) in vivo, we found that mPEG*‐b‐*P(Met/n‐PMA) had an excessive effect on weight control, as shown in Figure S21 (Supporting Information). mPEG*‐b‐*P(Met/n‐PMA) attenuated high‐fat diet (HFD)‐induced body weight gain compared to Lipo3000 and metformin. Therefore, we monitored the effect of mPEG*‐b‐*P(Met/n‐PMA) on the blood glucose levels. First, we tested the glucose tolerance test (GTT) and insulin tolerance test (ITT) before administration and compared them with the normal chow diet (NCD) diet. The GTT and ITT of the HFD, mPEG*‐b‐*P(Met/n‐PMA), and Lipo3000 improved, suggesting that the mice developed mild glucose and insulin resistance. After 14 d of administration, the GTT and ITT of mPEG*‐b‐*P(Met/n‐PMA) decreased compared with those of Lipo3000 and HFD, indicating that mPEG*‐b‐*P(Met/n‐PMA) ameliorated glucose and insulin resistance (Figure 6F and Figure S22, Supporting Information).

## 3 Conclusions

By designing, synthesizing, and characterizing novel mPEG*‐b‐*P(Met/n‐PMA), we found that mPEG*‐b‐*P(Met/n‐PMA) can formulate nucleic acids into stable nanomicelles via electrostatic and van der Waals interactions and that mPEG*‐b‐*P(Met/n‐PMA) can be selectively activated and disintegrated by CES in the liver. These nanomicelles can deliver nucleic acids to hepatocytes both in vitro and in vivo. This delivery strategy achieved efficient genome editing of PCSK9 and downregulation of serum LDL‐C. Notably, in addition to lipid‐lowering effects, this strategy has a dual effect on the regulation of blood glucose and weight. However, the mechanisms, pharmacokinetics, and pharmacodynamics of mPEG*‐b‐*P(Met/n‐PMA) in regulating blood glucose and weight remain unclear. Taken together, these data justify that further exploration of nucleic acid carriers with metformin is warranted. In addition, mPEG*‐b‐*P(Met/n‐PMA)‐mediated PCSK9 gene editing has the potential to ameliorate hyperlipidemia.

## 4 Experimental Section

### Materials

N‐succinimidyl methacrylate, AIBN, and dicyandiamide were obtained from Macklin (China). Ethylenediamine was purchased from Sigma‐Aldrich (USA), and bis(thiobenzoyl) disulfide (CPADN) was purchased from TCI (Japan). MeO‐PEG (mPEG) was obtained from RuixiBio (Beijing, China). The CCK8 kit was purchased from Beyotime (China). GFP mRNA, T7E1 endonuclease, and RNA Synthesis, and Purification kits were purchased from New England Biolabs (USA). Cas9‐mRNA was purchased from TriLink (USA). Antibodies against PCSK9 and LDLR were purchased from Abcam (Cambridge, UK). Lipofectamine 3000 was purchased from Thermo Fisher Scientific (USA). Penicillin–streptomycin solution (100×), trypsin, fetal bovine serum (FBS), and Dulbecco's modified Eagle medium (DMEM) were purchased from Gibco (USA). High‐purity (Milli‐Q) water with a resistivity of 18.2 MΩ cm was obtained from an inline Millipore RiOs/Origin water purification system.

### Instruments

The following instruments were used: a Bruker Avance III 600 MHz nuclear magnetic resonance (NMR) spectrometer (Bruker, Germany); a dynamic light scattering particle size analyzer (DLS, Malvern, UK), an ultraviolet‒visible (UV–vis) absorption spectrophotometer (UV1050, Techcomp, China); a Nicolet iS5 (Thermo Fisher Scientific, USA) for Fourier transform infrared (FTIR) spectroscopy; a Nanodrop2000 ultramicrospectrophotometer (Thermo Fisher Scientific, USA); an LC20 gel permeation chromatography (GPC, Shimadzu, Japan); a Nikon Eclipse Ti‐S inverted fluorescence microscope (Nikon Corporation, Japan); a field emission TEM (FEI, Netherlands); an NCF950 laser scanning confocal microscope (LSCM, Yongxin, China); an Illumina HiSeq2500 high throughput sequencer (Illumina, USA); and a flow cytometer (TCM, Beckman Coulter, USA).

### MD Simulation

MD simulations were performed using GROMACS 2018.4. Under constant temperature, pressure, and periodic boundary conditions, Amber14SB was used as the full atomic force field, and TIP3P was used as the water model. All the hydrogen bonds were restrained using the LinCS algorithm with an integral step size of 2 fs. The electrostatic interactions were calculated using the particle mesh Ewald. The cutoff of nonbonded energies was 10 Å and was updated every 10 steps. The v‐rescale temperature‐coupling method was used to control the simulated temperature to 298.15 K, and the Parrinello–Rahman method was used to control the analog pressure to 1 bar. To eliminate the effect of close contact between atoms, the energy of the mPEG*‐b‐*P(Met/n‐PMA)/RNA complex was minimized using the saddle point method. Then, 1 ns *NVT* and *NPT* equilibrium simulations were performed. Finally, an MD simulation of 100 ns was performed on the mPEG*‐b‐*P(Met/n‐PMA)/RNA complex, and the conformation was saved every 10 ps. Visualization of the simulation results was performed using VMD.

### Synthesis of N‐(2‐Aminoethyl)methacrylamide (Compound 2)

N‐Succinimidyl methacrylate (256 mg, 2 mmol) was dissolved in 10 mL of anhydrous tetrahydrofuran in an ice bath, and ethylenediamine (1.5 mL, 2.25 mmol) was added dropwise to the reaction. The resulting solution was then stirred at room temperature for 12 h. After the completion of the reaction, the white precipitate was removed by suction filtration, and the filtrate was concentrated by rotary evaporation. After drying under vacuum, a pale‐yellow sticky solid of N‐(2‐aminoethyl)methacrylamide (217 mg) was obtained with a yield of 84.7%.

### Synthesis of N‐(2‐(3‐Carbamimidoylguanidino)ethyl)methacrylamide (Compound 3)

N‐(2‐aminoethyl)methacrylamide (135.9 mg, 1.05 mmol) and dicyandiamide (72 mg, 1.15 mmol) were dissolved in 10 mL of a 2% hydrochloric acid solution. The reaction temperature was increased to 90 °C, and the mixture was stirred for 3 h. Then, it was concentrated via rotation‐evaporation, and N‐(2‐(3‐carbamimidoylguanidino)ethyl)methacrylamide was obtained in 80.3% yield.

### Synthesis of mPEG‐CPADN (Compound 5)

MeO‐PEG‐NH_2_ (100 mg, 50 µmol) and NHS‐modified CPADN (300 mg, 1 mmol) were dissolved in 10 mL of dry DCM and stirred overnight at room temperature. The solution was then recrystallized from cold diethyl ether thrice and centrifuged to obtain a pink precipitate. Finally, the precipitate was dried by lyophilization to form a pink powder.

### Synthesis of mPEG*‐b‐*P(Met/n‐PMA) (Compound 7)

mPEG‐CPADN (50 mg, 25 µmol), *n*‐propyl methacrylate (39 µL, 25 µmol), N‐(2‐(3‐carbamimidoylguanidino)ethyl)methacrylamide (79 mg, 25 µmol), and AIBN (0.85 mg, 5 mmol) were dissolved in dried dimethyl sulfoxide (DMSO) and sealed in a flask under nitrogen. The reaction proceeded at 70 °C for 24 h. Then, the solution was cooled to room temperature, extracted with methyl tertiary‐butyl ether, and dialyzed against water for 1 d. The final product, mPEG*‐b‐*P(Met/n‐PMA) (23.6 mg, 14.5% yield), was obtained via freeze‐drying.

### Synthesis of mPEG*‐b‐*P(Met)

mPEG‐CPADN (50 mg, 25 µmol), N‐(2‐(3‐carbamimidoylguanidino)ethyl)methacrylamide (79 mg, 25 µmol), and AIBN (0.85 mg, 5 mmol) were dissolved in dried DMSO and sealed in a flask under nitrogen. The reaction proceeded at 70 °C for 24 h. Then, the solution was dialyzed against water for 1 d. The final product, mPEG*‐b‐*P(Met/n‐PMA) (23.6 mg, 11.6% yield), was obtained via freeze‐drying.

### Preparation of mPEG*‐b‐*P(Met/n‐PMA)/Cas9‐mRNA/sgPCSK9

A solution of mPEG*‐b‐*P(Met/n‐PMA) was mixed with Cas9‐mRNA and sgPCSK9 (weight ratio of 2:1) at a Gu^+^/PO_3_^4−^ molar ratio of 3:1 in HEPES buffer (10 × 10^−3^ m, pH 7.4) and incubated at room temperature for 30 min. The resulting complex was denoted as mPEG*‐b‐*P(Met/n‐PMA)/Cas9‐mRNA/sgPCSK9. The size, size distribution, and morphology of the mPEG*‐b‐*P(Met/n‐PMA)/Cas9‐mRNA/sgPCSK9 cells were measured via DLS and TEM.

### Polyacrylamide Gel Electrophoresis Assay

The RNA‐binding ability of mPEG*‐b‐*P(Met/n‐PMA) was evaluated using agarose gel electrophoresis. The solution of mPEG*‐b‐*P(Met/n‐PMA) was mixed with sgPCSK9 at different molar ratios of Gu^+^/PO_3_^4−^ in HEPES buffer (10 × 10^−3^ m, pH 7.4) and incubated for 30 min. The incubated mixture was electrophoresed on a 15% polyacrylamide gel at 110 V in TBE for 2 h. Gel images were obtained using a Molecular Image.

### Stability of mPEG*‐b‐*P(Met/n‐PMA) in Esterase

One milliliter of the mPEG*‐b‐*P(Met/n‐PMA) solution was added to a dialysis bag (cutoff Mn = 10 000 Da) containing esterase or denatured esterase (30 U mL^−1^). The mixture was then kept at 37 °C under constant shaking at different times.

### Stability and In Vitro Release of mPEG*‐b‐*P(Met/n‐PMA)/Cas9‐mRNA/sgPCSK9

The mPEG*‐b‐*P(Met/n‐PMA)/Cas9‐mRNA/sgPCSK9 solution was added to a dialysis bag (cut‐off Mn = 10 000 Da) with or without esterase (30 U mL^−1^). The mixtures were maintained at different temperatures under constant shaking for 4 h.

### Cell Culture

HepG2, HEK293T, and HeLa cells were purchased from ATCC (USA). The cells were cultured in 10% FBS‐supplemented DMEM, 100 U mL^−1^ penicillin, and 100 mg mL^−1^ streptomycin.

### Transfection

HepG2 cells were seeded on six‐well cell culture clusters at a density of 60%. Next, Opti‐MEM‐reduced serum medium containing Cas9‐mRNA/sgPCSK9, mPEG*‐b‐*P(Met/n‐PMA)/Cas9‐mRNA/sgPCSK9, and Lipo3000/Cas9‐mRNA/sgPCSK9 was added to the cells and incubated for 48 h at 37 °C with 5% CO_2_.

### Confocal Microscopy Imaging

GFP and its mRNA were used to express green fluorescence. Cyanine 5 (Cy5) was used to label sgPCSK9 (cy5‐sgPCSK9), and 4′,6‐diamidino‐2‐phenylindole (DAPI) was used to label nuclei. HepG2, HeLa, HEK293, and A549 cells were seeded in confocal culture dishes at a density of 60% and transfected as described above. The medium was then removed, and the cells were washed with PBS thrice and observed under a confocal microscope.

### Flow Cytometry Assays

The transfected cells were rinsed thrice with PBS, trypsinized, and collected. The cells were then resuspended in PBS and analyzed using a flow cytometer.

### CCK8 Assay

HepG2 cells were seeded in a 96‐well plate at densities of 4000 and 5000 cells per well and cultured for 24 h. The cells were then exposed to a series of concentrations of HEPES buffer, metformin, mPEG*‐b‐*P(Met/n‐PMA), and Lipo3000 for 48 or 72 h. Subsequently, the cells were washed thoroughly with PBS, and the CCK‐8 solution was added to each well to a concentration of 10%. The plate was incubated at 37 °C, and the absorbance was measured at 450 nm.

### T7 Endonuclease I (T7EI) Mutation Detection

T7EI mutated HepG2 cells at a density of 60% cells mL^−1^ were seeded into six‐well plates. The cells were then transfected with Cas9‐mRNA/sgPCSK9 and mPEG*‐b‐*P(Met/n‐PMA)/Cas9‐mRNA/sgPCSK9 and cultured at 37 °C for 48 h. Genomic DNA was isolated and harvested using a genomic DNA extraction kit (Tian Gen, China), according to the manufacturer's instructions. Targeted DNA fragments at the PCSK9 locus were amplified from the genomic DNA of wild‐type or transfected cells. A pair of optimized PCR primers was designed, and the annealed PCR products were 396 bp and ranged from upstream to downstream at the targeted site.

Primer:

PCSK9 sense primer: 5′ ‐ACCCACCTCCTCACCTTTCC‐3′

PCSK9 antisense primer: 5′‐CCCTGACCTCGTGTTTCCTC‐3′

After amplification, 5 µL amplicon was diluted with 1.1 µL 10× T7E1 buffer and 4.4 µL nuclease‐free water. The amplicon was denatured at 100 °C in boiling water and allowed to cool naturally to room temperature for rehybridization. Next, 0.5 µL of T7E1 was added to the tube and incubated at 37 °C for 30 min. Then, 1 µL of proteinase K was added and mixed well. Samples were electrophoresed using 5% polyacrylamide gel.

### Western Blot Assay

Proteins from cell and tissue lysates were extracted using RIPA buffer. A total of 20 µg of protein was used for western blotting. The following primary antibodies were used: anti‐PCSK9, anti‐LDL‐R, and anti‐*β*‐actin. The primary antibody was used at a 1:8000 dilution. The antibodies were validated by molecular mass analysis using a positive control sample.

### Measurement of Intracellular Cholesterol

Transfected HepG2 cells in six‐well plates were cultured for 12 h in serum‐free medium with or without 50 µg mL^−1^ LDL. Cells were then washed twice in PBS, and intracellular lipids were extracted in isopropanol, dried under vacuum, and TC was measured using an enzymatic assay and normalized to total cell protein as determined by the modified Lowry assay. For morphological examination, HepG2 cells were fixed for 30 min with 5% formalin solution in PBS, stained with Oil Red O for 30 min, and counterstained with hematoxylin for 5 min. Finally, the cells were examined under a light microscope.

### Animals

All animal procedures were performed in accordance with the National Institute of Health Guidelines for the Care and Use of Laboratory Animals. C57BL/6J mice and an ethical approval statement (2021015) were provided by the Experimental Animal Center of Chongqing Medical University. Age‐matched (6–8 weeks old) male wild‐type (WT) C57BL/6 mice received either a NCD or a HFD containing 60 kcal% fat for 12 weeks (*n* = 6). The mice were maintained under a constant 12‐h light/dark cycle with unrestricted access to food and water.

### Biodistribution In Vivo

The mPEG*‐b‐*P(Met/n‐PMA) and mPEG*‐b‐*P(Met/n‐PMA)/Cy5‐sgPCSK9 nanoparticles were prepared as described above. The mPEG*‐b‐*P(Met/n‐PMA) nanoparticles were stained with Cy5. The nanoparticles were administered via intravenous injection and tracked for 24 h using an in vivo Imaging system.

### Tests of Biochemical Parameters

Male C57 mice (6–8 weeks) were randomized for treatment with saline, mPEG*‐b‐*P(Met/n‐PMA), mPEG*‐b‐*P(Met/n‐PMA)/Cas9‐mRNA/sgPCSK9, Cas9‐mRNA/sgPCSK9, or Lipo3000/Cas9‐mRNA/sgPCSK9. Cas9‐mRNA and sgPCSK9 were used in a weight ratio of 2:1. These formulations were administered intravenously every 7 d. Immediately after euthanasia of the animals 28 d after receiving saline, saline, mPEG*‐b‐*P(Met/n‐PMA), mPEG*‐b‐*P(Met/n‐PMA)/Cas9‐mRNA/sgPCSK9, Cas9‐mRNA/sgPCSK9, and Lipo3000/Cas9‐mRNA/sgPCSK9 were administered and ELISA was performed to evaluate PCSK9 protein, LDL‐C, HDL‐C, TC, TG, ALT, AST, BUN, S‐Cr, and triglyceride levels in plasma samples.

### Histological Analysis

After being injected with saline, mPEG*‐b‐*P(Met/n‐PMA), mPEG*‐b‐*P(Met/n‐PMA)/Cas9‐mRNA/sgPCSK9, Cas9‐mRNA/sgPCSK9, and Lipo3000/Cas9‐mRNA/sgPCSK9, they were sacrificed, and the major organs, including the liver, heart, spleen, lung, and kidney, were extracted. Then, the tissues were fixed with 10% formalin solution, embedded in paraffin, and sectioned to ≈5 µm. The sections were stained with H&E.

### GTTs and ITTs

For GTTs, the mice were fasted for 12 h and then intraperitoneally injected with 1 g kg^−1^ body weight d‐glucose. For ITTs, the mice were fasted for 4 h and were intraperitoneally injected with 0.14 units kg^−1^ body weight insulin. Blood glucose levels were measured at 0, 15, 30, 60, and 120 min in tail vein blood using a GA‐7 Advantage glucometer (Sannuo).

### Statistical Analysis

Statistical analyses were performed using SPSS software (Version 18.0; IBM Corp., Armonk, NY). The results are depicted as the mean ± SD, and statistical tests are indicated in the figures. Differences were considered statistically significant at *P* < 0.05. Experimental data were compared using one‐way analysis of variance (ANOVA).

## Conflict of Interest

The authors declare no conflict of interest.

## Supporting information

Supporting Information

## Data Availability

The data that support the findings of this study are available from the corresponding author upon reasonable request.
