# Supplementary material for: CES1‐Triggered Liver‐Specific Cargo Release of CRISPR/Cas9 Elements by Cationic Triadic Copolymeric Nanoparticles Targeting Gene Editing of PCSK9 for Hyperlipidemia Amelioration
Source: Adv Sci (Weinh). 2023 Apr 21;10(19):2300502. doi: 10.1002/advs.202300502 (PMC10323666; doi:10.1002/advs.202300502)
Supplement: Supplementary file 1 — Supporting Information [file ADVS-10-2300502-s001.pdf]

ADVANCED SCIENCE
Open Access

Supporting Information

for *Adv. Sci.*, DOI 10.1002/advs.202300502

CES1-Triggered Liver-Specific Cargo Release of CRISPR/Cas9 Elements by Cationic Triadic Copolymeric Nanoparticles Targeting Gene Editing of PCSK9 for Hyperlipidemia Amelioration

Yunfei Zhao, Yun Li, Fan Wang, Xuelan Gan, Tianye Zheng, Mengyue Chen, Li Wei, Jun Chen and Chao Yu**

Supporting information

CES1-triggered liver-specific cargo release of CRISPR/Cas9 elements by cationic triadic copolymeric nanoparticles targeting gene editing of PCSK9 for hyperlipidemia amelioration

Yunfei Zhao[#], Yun Li[#], Fan Wang[#], Xuelan Gan, Tianye Zheng, Mengyue Chen, Li Wei, Jun Chen, Chao Yu**

Y. F. Zhao, Dr. F. Wang, X.L. Gan, T. Y. Zheng, F. L. Wang, Dr. J. Chen, Prof. C. Yu

*Corresponding author E-mail: Jun Chen: 191035@cqmu.edu.cn; Chao Yu: yuchao@cqmu.edu.cn

Y. F. Zhao, Dr. F. Wang, X.L. Gan, T. Y. Zheng, F. L. Wang, Dr. J. Chen, Prof. C. Yu

Chongqing Key Laboratory for Pharmaceutical Metabolism Research, Chongqing 400016, P.R. China

Y. F. Zhao, Dr. F. Wang, X.L. Gan, T. Y. Zheng, F. L. Wang, Dr. J. Chen, Prof. C. Yu

Chongqing pharmacodynamic evaluation engineering technology research center, Chongqing 400016, P.R. China

Dr. Y. Li, Dr. M. Y Chen, L. Wei,

Centre for Lipid Research & Key Laboratory of Molecular Biology for Infectious Diseases (Ministry of Education), Institute for Viral Hepatitis, Department of Infectious Diseases, the Second Affiliated Hospital, Chongqing Medical University, Chongqing 400016, P.R. China

# Yunfei Zhao, Yun Li, Fan Wang contributed equally to this work.

Result

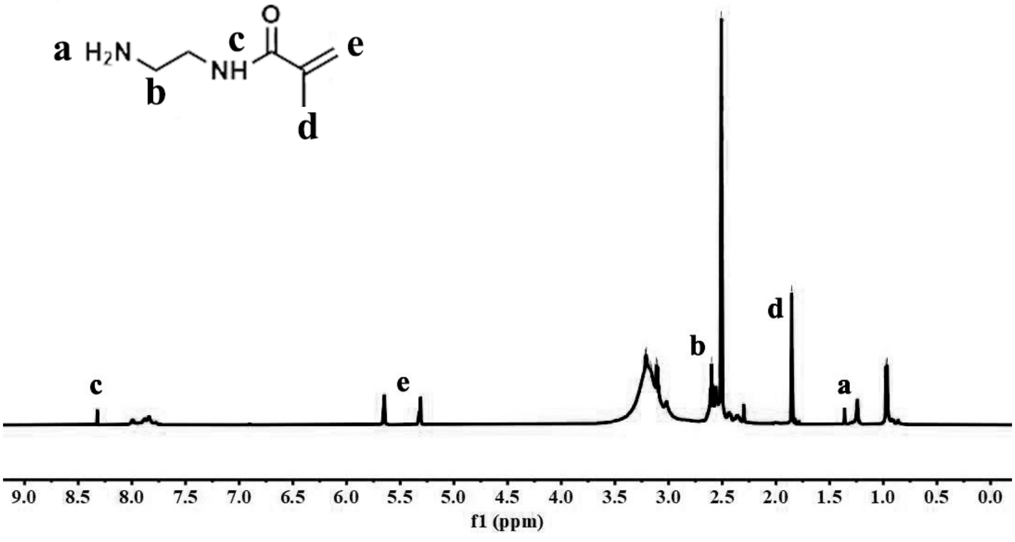

Fig. S1. ^{1}H NMR spectrum of N-(2-aminoethyl)methacrylamide (Compound 2, CD_3Cl, 600 MHz).

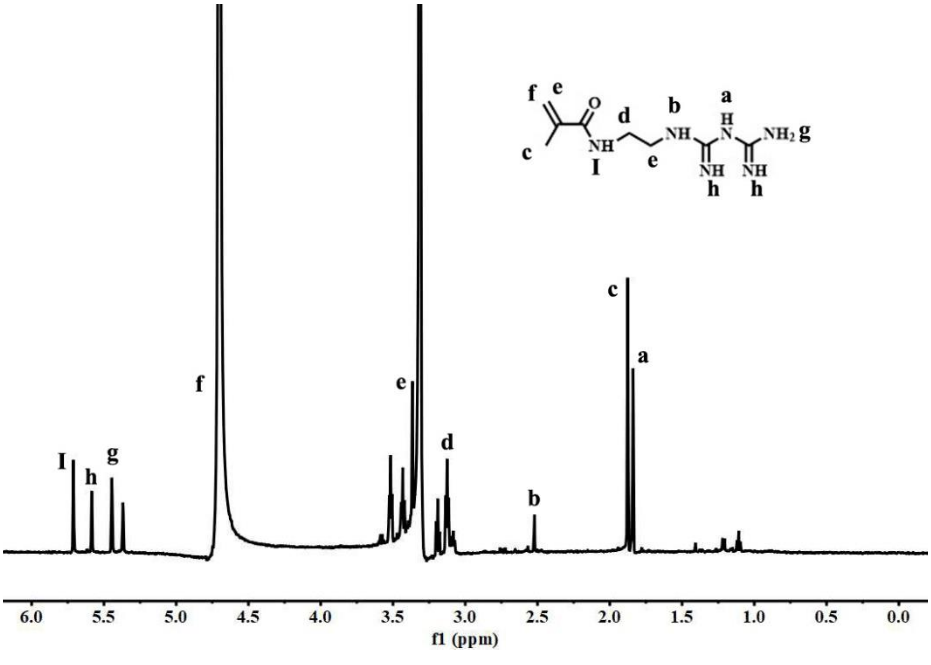

Fig. S2. ^{1}H NMR spectrum of N-(2-(3-carbamimidoylguanidino)ethyl)methacrylamide (Compound 3, D_2O, 600 MHz).

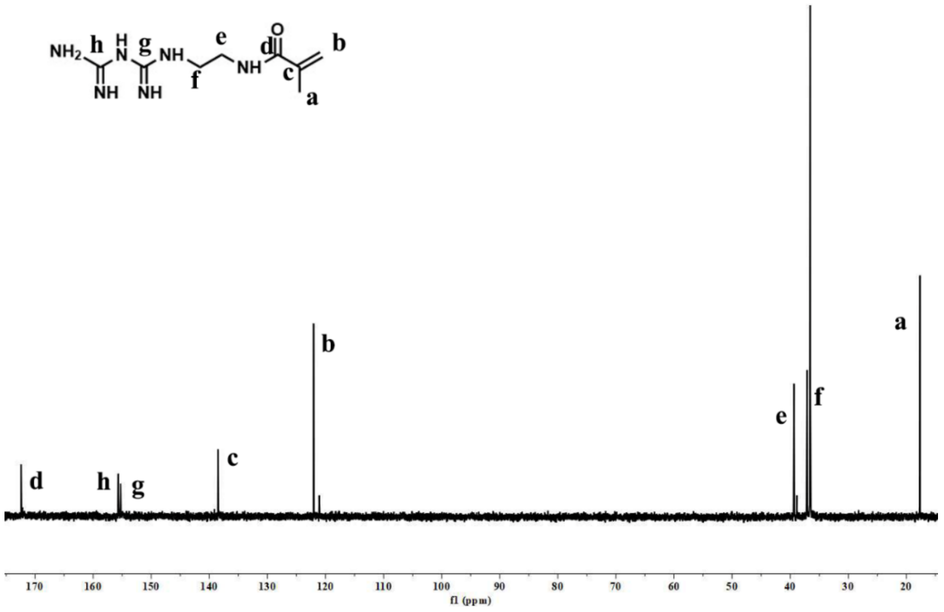

Fig. S3. ^{13}C NMR spectrum of N-(2-(3-carbamimidoylguanidino)ethyl)methacrylamide (Compound 3, D_2O, 600 MHz).

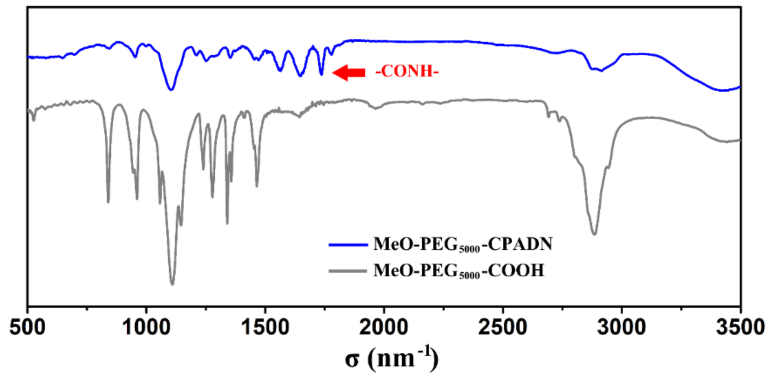

Fig. S4. Infrared spectroscopy (IR) spectra of MeO-PEG_{5000}-CPADN (blue) and MeO-PEG_{5000}-COOH (gray).

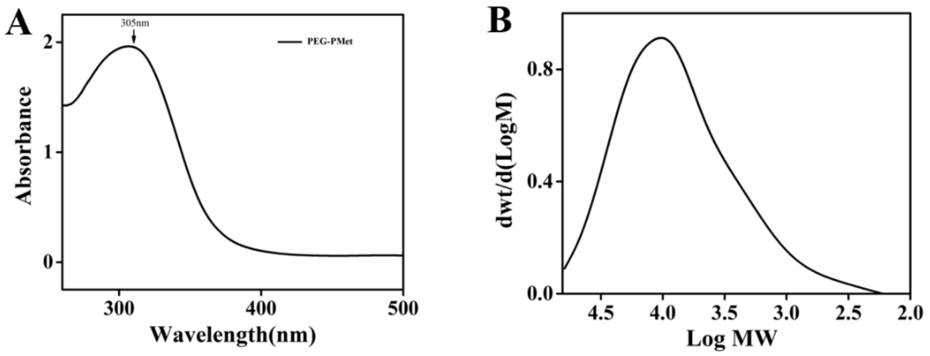

Fig. S5. (A) Ultraviolet and visible spectrum absorption (UV) and (B) Gel Permeation Chromatography (GPC, right) image of mPEG-b-P(Met/n-PMA).

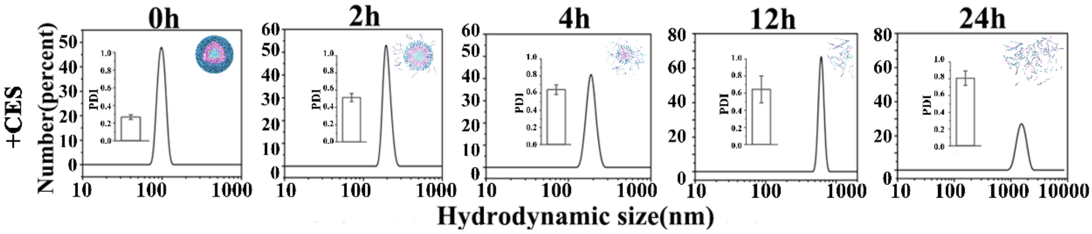

Fig. S6. DLS and PDI images of mPEG-*b*-P(Met/n-PMA) assemblies under conditions with 30 U/mL CES. Bars represent the mean ± SD (n = 3).

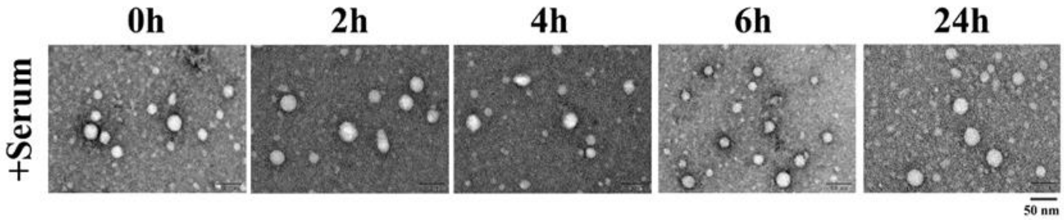

Fig. S7. TEM image of mPEG-*b*-P(Met/n-PMA) under conditions with mouse serum in different times. Scare bar: 50 nm

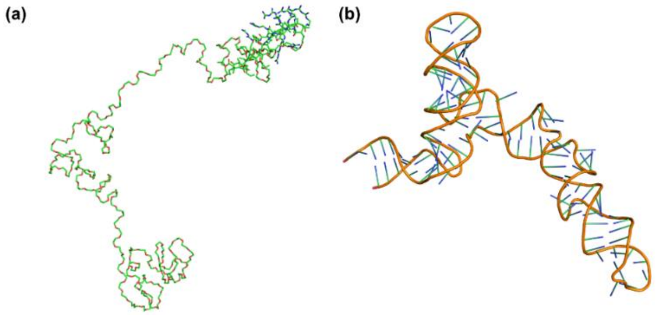

Fig. S8. Three-dimensional structural models of (a)PEG-PMet and (b)mRNA.

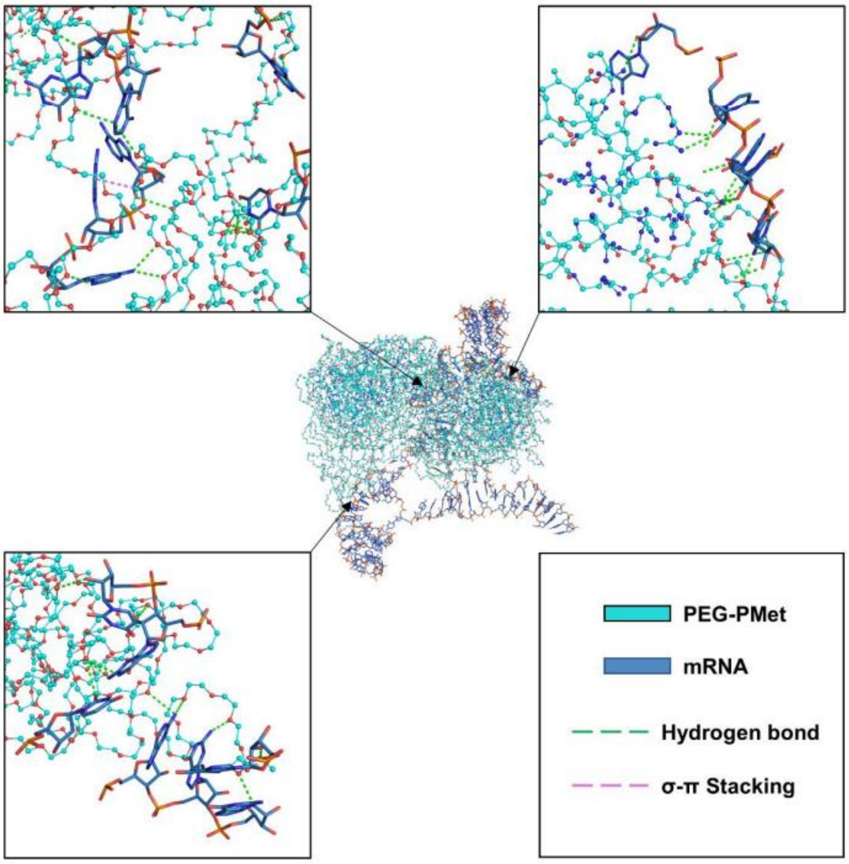

Fig. S9. Snapshot highlights the stabilizing effect of PEG-PMet (green stick) through its interaction with mRNA (blue). The interaction includes hydrogen bonds (green dashed line) and σ-π stacking (pink).

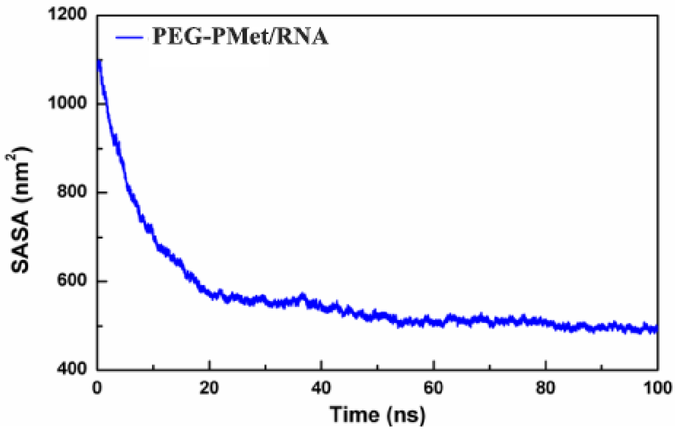

Fig. S10. Variations in solvent accessible surface area (SASA) in the mPEG-b-P(Met/n-PMA)/RNA complex.

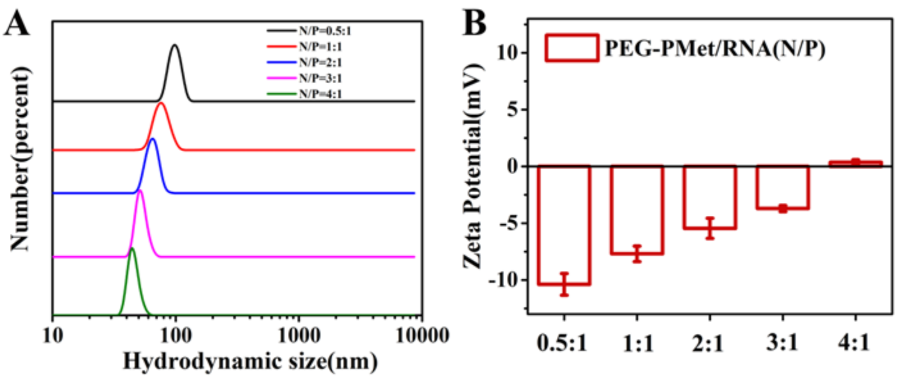

Fig. S11. DLS (A) and zeta potential (B) image of mPEG-*b*-P(Met/n-PMA)/RNA under different N/P ratios. Bars represent the mean ± SD (n = 3).

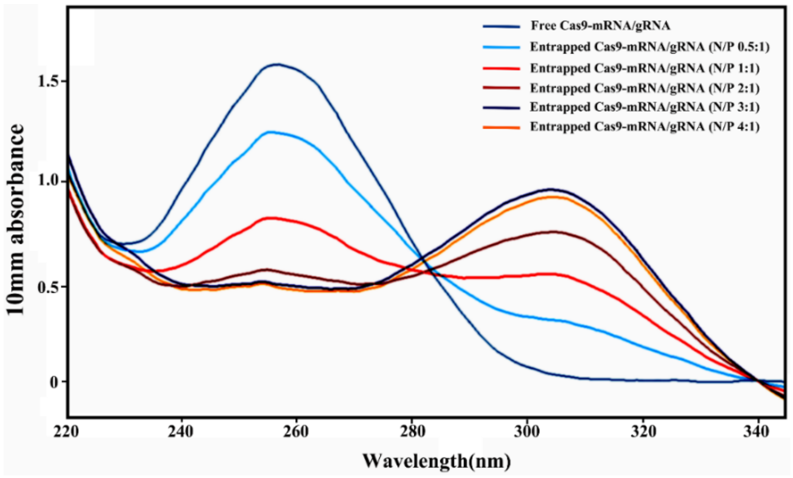

Fig. S12. Detection of RNA binding ability of different mole ratios of mPEG-b-P(Met/n-PMA) by UV spectroscopy (260 nm: RNA, 305 nm: mPEG-b-P(Met/n-PMA))

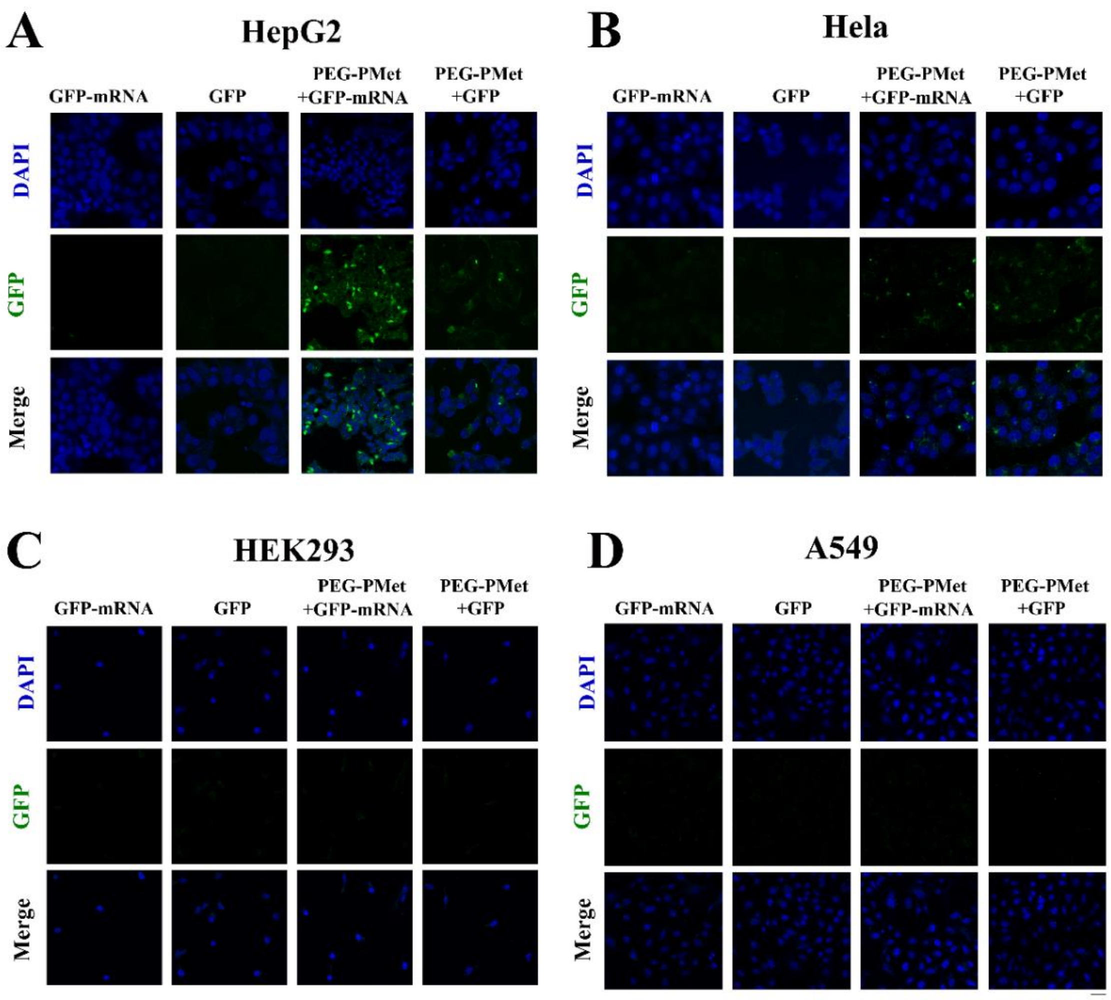

Fig. S13. CLSM analysis of GFP, GFP-mRNA, mPEG-*b*-P(Met/n-PMA)/GFP, and mPEG-*b*-P(Met/n-PMA)/GFP-mRNA cellular uptake and transcript accumulation in different cells, including (A) HepG2, (B) HeLa, (C) HEK293, and (D) A549 cells. Green: GFP, Blue: Nucleus. Scale Bar: 50 μm.

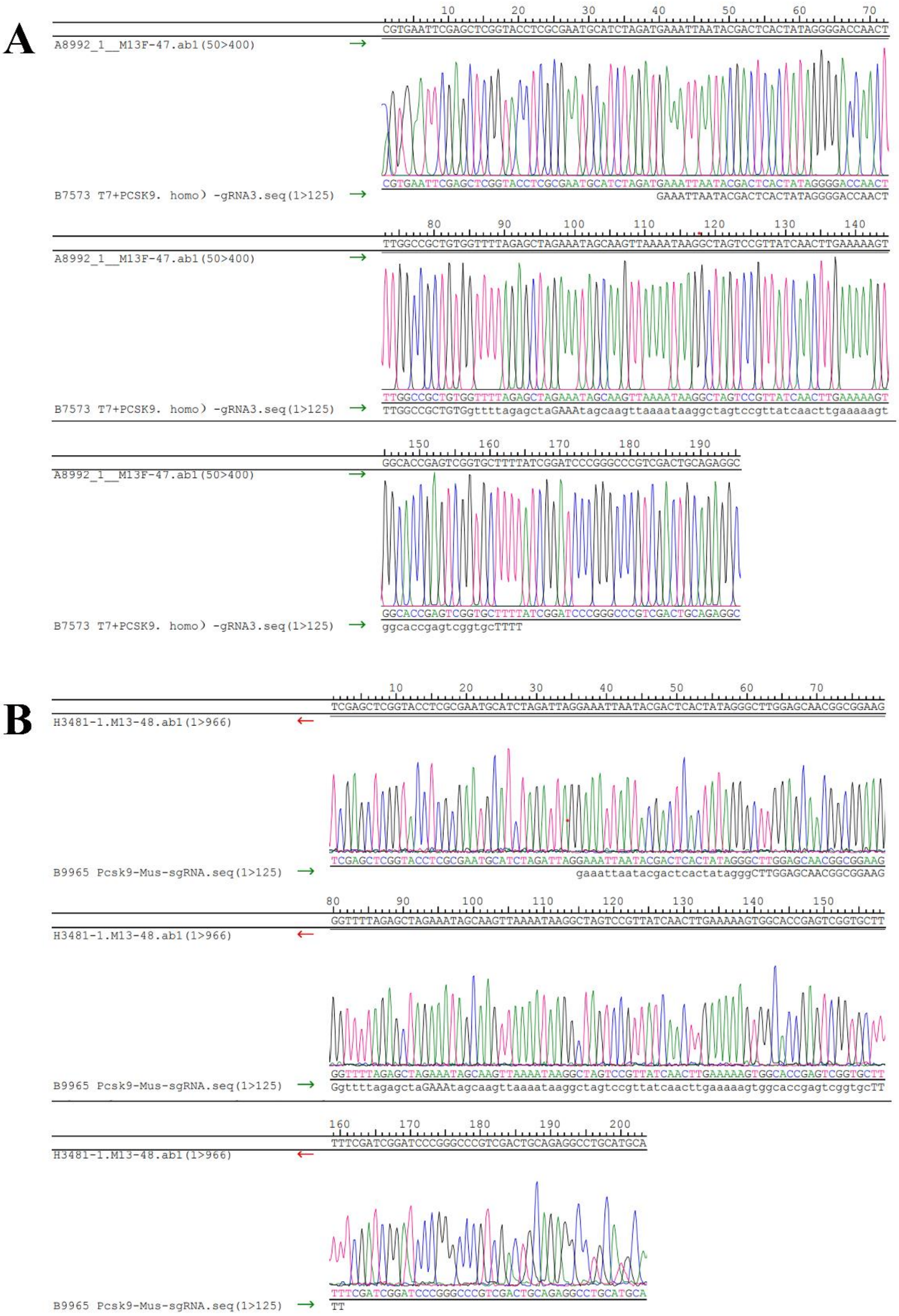

Fig. S14. (A) Sanger sequencing of human and (B) murine sgPCSK9. sgPCSK9 was synthesized by transcription in vitro and evaluated with Sanger sequencing

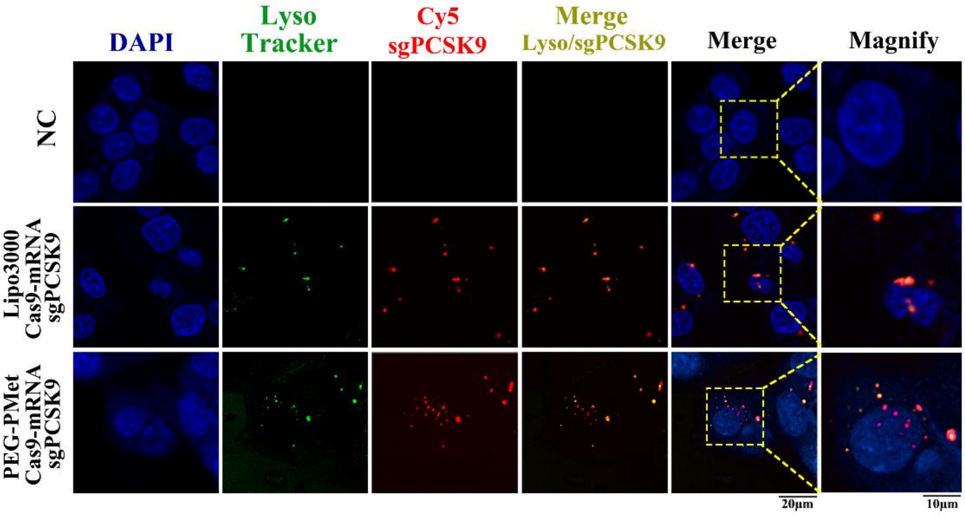

Fig. S15. CLSM analysis of lysosomal escape capacity in HepG2 cells. The cells were transfected with different formulations, including negative control (NC), Lipo3000/Cas9-mRNA/sgPCSK9, and mPEG-b-P(Met/n-PMA)/Cas9-mRNA/sgPCSK9. Scale Bar: 20 μm and 10 μm of magnification.

ID	Sequence	Mismatches	Locus
sgPCSK9	GGACCAACTTTGGCCGCTGTG-TGG	0	Chr1
1	GCACCATCTTTGGTCCCTGTG-CGG	4	Chr9
2	GGACCCACTGTGGACGCTGTG-CGG	3	Chr2
3	GCACAAACTGTGACCGCTGTG-GGG	4	Chr18
4	GGGCCAGCTTTGGCAACTGTG-AGG	4	Chr8
5	GGAACCAATTTTTGCCTCTGTGAGG	4	Chr2
6	GGAGGAACTTTGGCCAGTGTG-GGG	4	Chr5
7	GGACCAACTTAGGTTCCTGTGGGG	4	Chr10
8	GGACCACGTCTGGCAGCTGTGTGG	4	Chr19

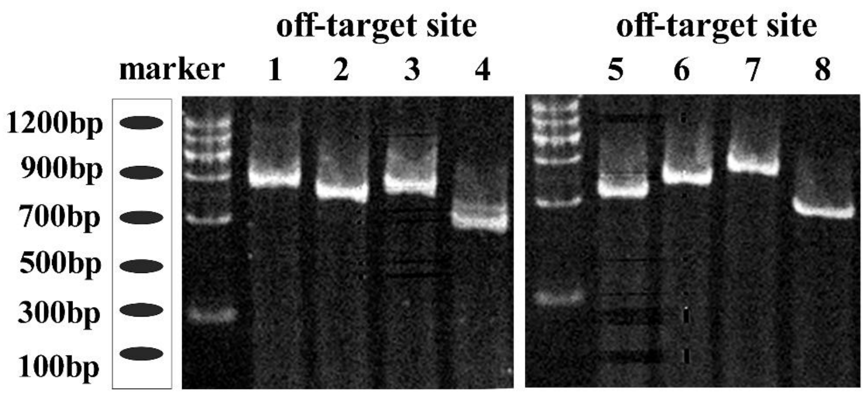

Fig. S16. No off-target editing was detected after treatment with mPEG-b-P(Met/n-PMA)/Cas9-mRNA/sgPCSK9 targeted gene PCSK9. The top eight potential off-target sites were amplified using PCR and analyzed using T7EI assay.

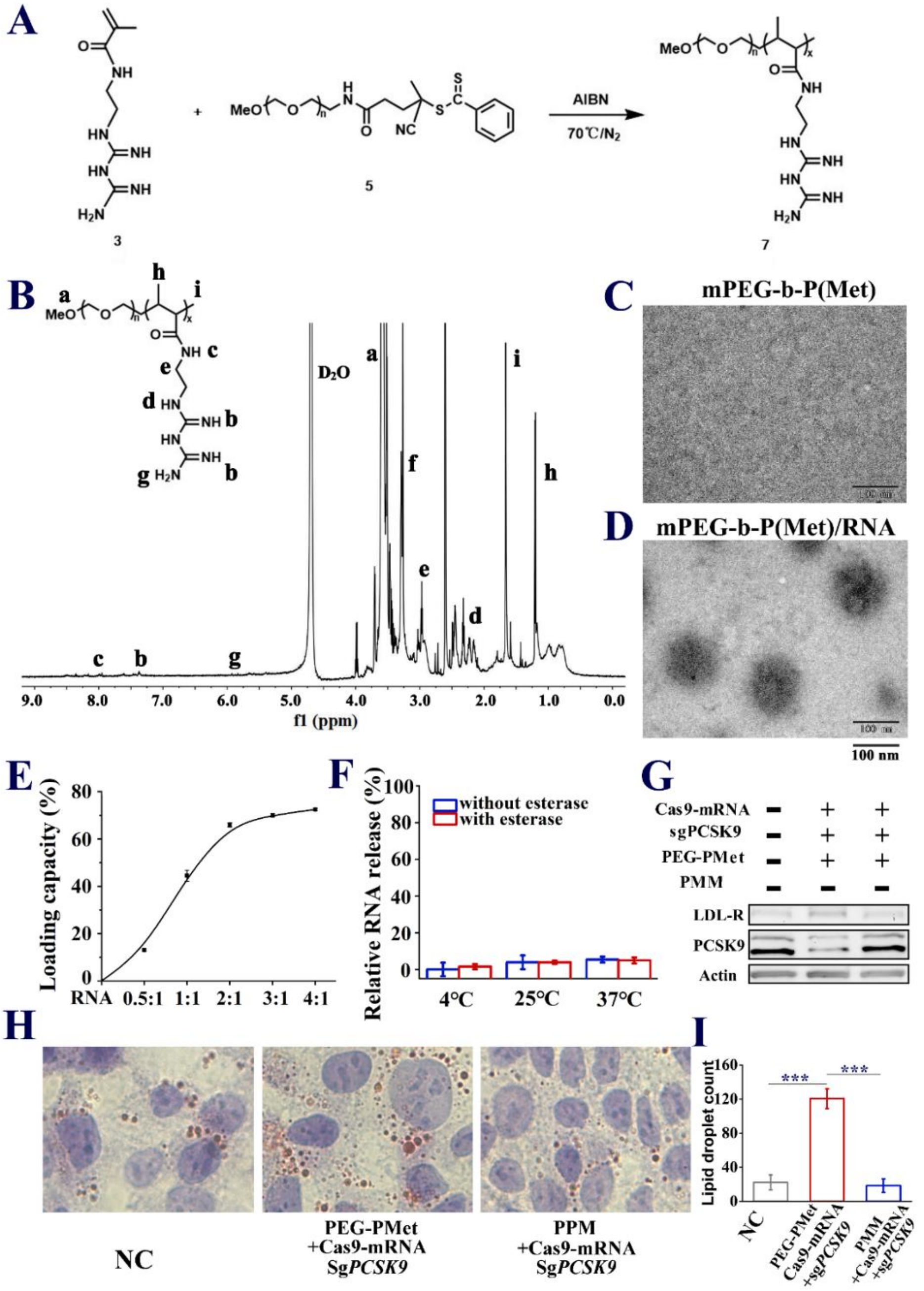

Fig. S17. (A) Synthesis of mPEG-b-P(Met). (B) 1H-NMR spectrum of mPEG-b-P(Met) (D_2O, 600 MHz). (C) TEM image of mPEG-b-P(Met/n-PMA) and (D) mPEG-b-P(Met/n-PMA)/RNA. Scale bar: 100 nm. (E) Loading capacity of mPEG-b-P(Met) for RNA in mass. Bars represent the mean ± SD (n = 3). (F) RNA release from mPEG-b-P(Met) following esterase treatment as determined by UV spectroscopy. Bars represent the mean ± SD (n = 3). (G) PCSK9 and LDL-R protein levels in HepG2 cells were examined via western blotting analysis after treatment with mPEG-b-P(Met/n-PMA)/Cas9-mRNA/sgPCSK9, and mPEG-b-P(Met)/Cas9-mRNA/sgPCSK9. (H)(I) Morphological examination and (N) lipid counts of HepG2 cells after transfection with mPEG-b-P(Met/n-PMA)/Cas9-mRNA/sgPCSK9, and mPEG-b-P(Met)/Cas9-mRNA/sgPCSK9. (Oil Red O staining, original magnification ×400, analyzed by Image-Pro Plus). ***P < 0.001, **P < 0.01, Bars represent the mean ± SD (n = 3).

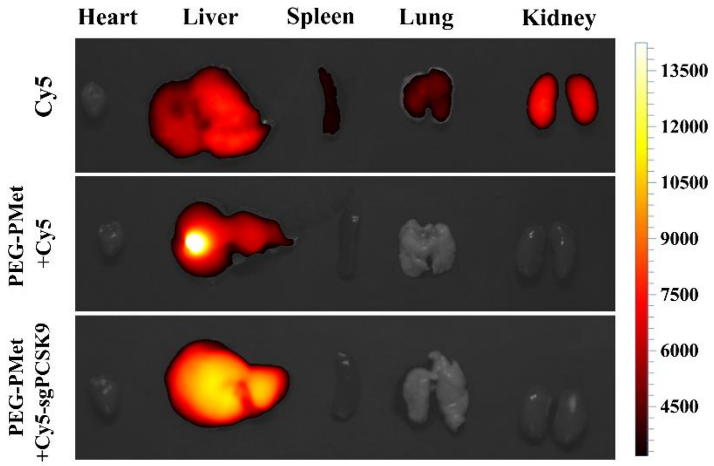

Fig. S18. Ex vivo fluorescence image of excised organs after injection of Cy5, mPEG-b-P(Met/n-PMA)/Cy5, and mPEG-b-P(Met/n-PMA)/Cy5-sgPCSK9 for 0.5 h.

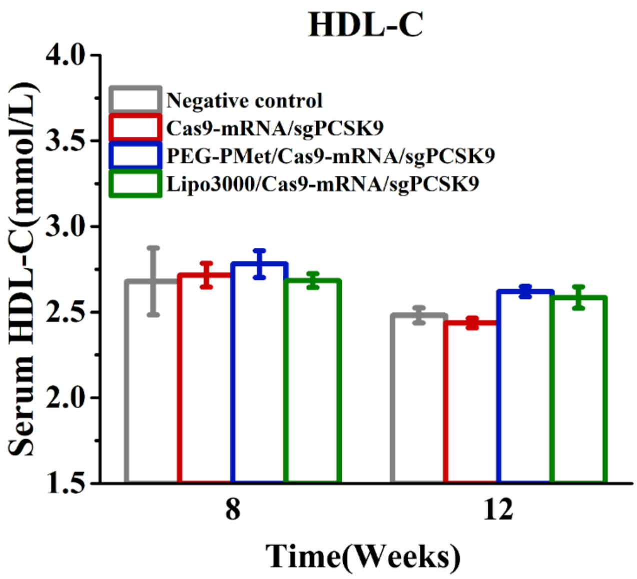

Fig. S19. Serum levels of HDL-C in mice 14 d or 42 d after administration of mPEG-b-P(Met/n-PMA)/Cas9-mRNA/sgPCSK9 and those in the control groups (n=3 mice for each group)

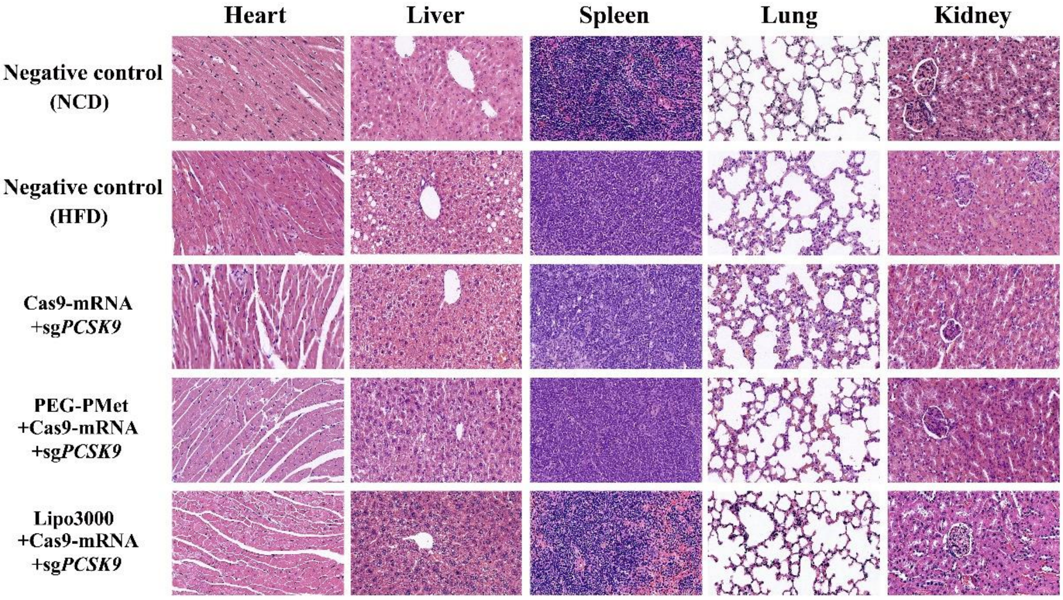

Fig. S20. HE staining images of major organs were collected from mice treated with mPEG-b-P(Met/n-PMA)/Cas9-mRNA/sgPCSK9 and from the control groups.

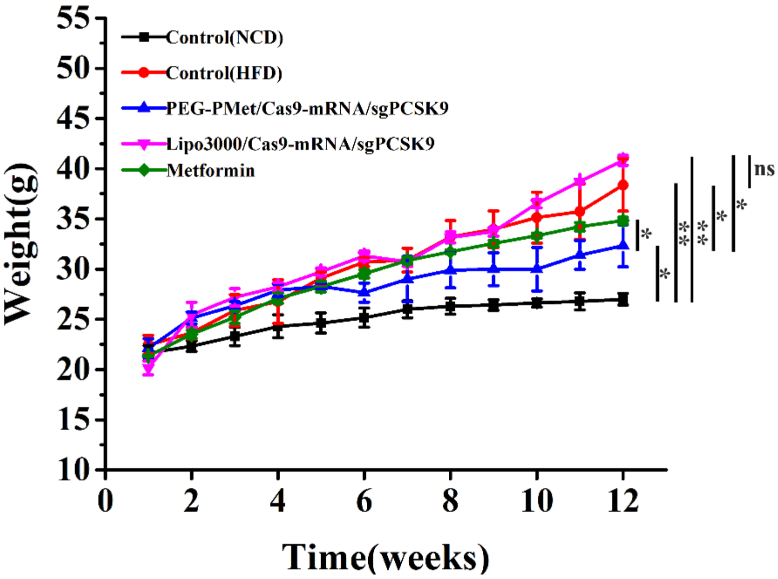

Fig. S21. Body weight changes in C57BL/6 mice treated with different diets and treatments. ***P < 0.001, **P < 0.01, Bars represent the mean ± SD (n = 3). P-values are calculated using one-way ANOVA with Bonferroni correction

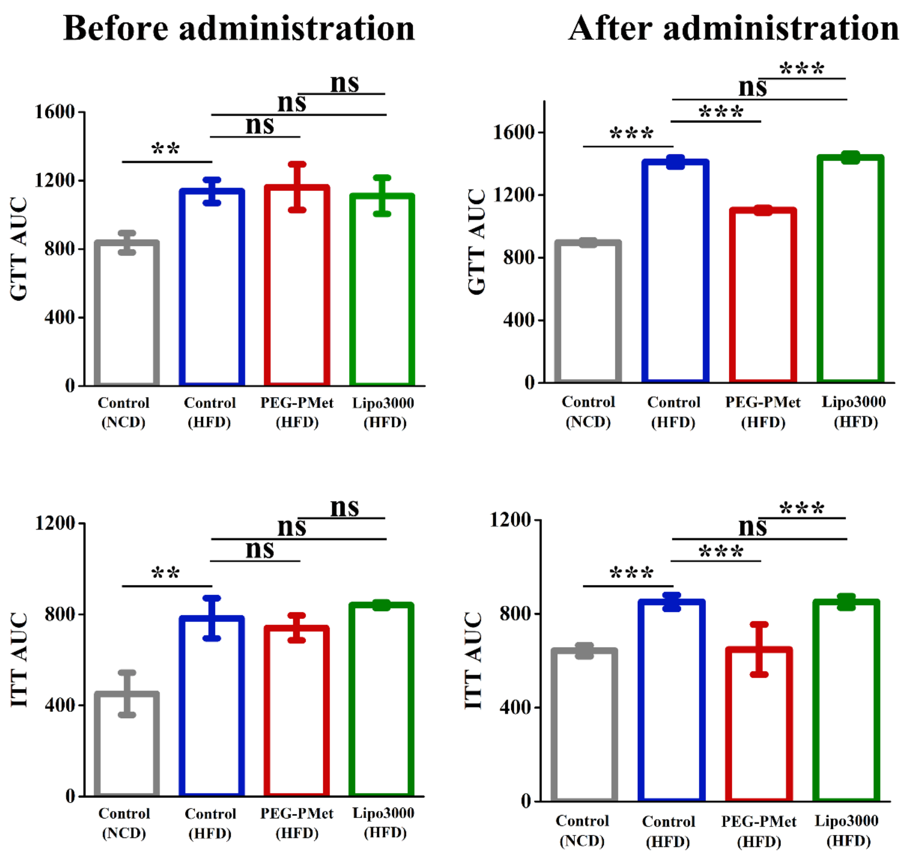

Fig. S22. Quantification of the area under the curve (AUC) of glucose tolerance tests (GTTs, up) and insulin tolerance tests (ITTs, down) in HFD-fed mice before, during, and after the administration of mPEG-*b*-P(Met/n-PMA) and Lipo3000 (n=3). ***P < 0.001, **P < 0.01, Bars represent the mean ± SD (n = 3). P-values are calculated using one-way ANOVA with Bonferroni correction

Table 1. Sequences of oligonucleotides

Oligo names	Sequences (5' to 3')
**sgPCSK9(Homo)**	GGACCAACTTTGGCCGCTGTG TGG
**sg*Pcsk9*(Mus)**	CTTGGAGCAACGGCGGA AGG
**F-PCSK9**	ACCCACCTCCTCACCTTTCC
**R-PCSK9**	CCCTGACCTCGTGTTTCCTC
**F-ADAMTSL2 (1)**	GAAAGGCCCTGACTGCTGA
**R-ADAMTSL2**	CAGACCACCTGTCACTCCTCA
**F-LOC110121204 (2)**	GAGCTGCCTCCTGCTTGACA
**R-LOC110121204**	CGGCTGAGACTCCCAGAAATG
**F-NFATC1 (3)**	TTGGGCTCTTTTGTCTGAACC
**R-NFATC1**	TTGTCGTCGTGGGAACTCG
**F-LOC12746032 (4)**	CCTAAATGATGTGACCCATTCTG
**R-LOC12746032**	GGCCTCAGTCTGTCAGTGGTAA
**F-ATAD2B (5)**	TCCCTGATGTGCCCTAT
**R-ATAD2B**	TGTACTGTTTGCGGTCCCAC
**F- ARHGAP26 (6)**	ACTATTATGGGTGGGAGGAA
**R-ARHGAP26**	TAGCTGGGCATGGTGG
**F-FRMD4A (7)**	ATTATCGGACACGGATGA
**R-FRMD4A**	TTCGCTGCCTGAGATT
**F-PEPD (8)**	CTGGAGCCTGGCACAGA
**R-PEPD**	GGAACCTCAAGGAGCAG

Fig. S23. Oligonucleotide sequences used in this study.
